# Small-RNA analysis of pre-basic mother plants and conserved accessions of plant genetic resources for the presence of viruses

**DOI:** 10.1371/journal.pone.0220621

**Published:** 2019-08-07

**Authors:** Minna-Liisa Rajamäki, Anne Lemmetty, Jaana Laamanen, Elina Roininen, Archana Vishwakarma, Janne Streng, Satu Latvala, Jari P. T. Valkonen

**Affiliations:** 1 University of Helsinki, Department of Agricultural Sciences, Helsinki, Finland; 2 Natural Resources Institute Finland (Luke), Jokioinen, Finland; 3 Natural Resources Institute Finland (Luke), Jyväskylä, Finland; Oklahoma State University, UNITED STATES

## Abstract

Pathogen-free stocks of vegetatively propagated plants are crucial in certified plant production. They require regular monitoring of the plant germplasm for pathogens, especially of the stocks maintained in the field. Here we tested pre-basic mother plants of *Fragaria*, *Rubus* and *Ribes* spp., and conserved accessions of the plant genetic resources of *Rubus* spp. maintained at research stations in Finland, for the presence of viruses using small interfering RNA (siRNA) -based diagnostics (VirusDetect). The advance of the method is that unrelated viruses can be detected simultaneously without resumptions of the viruses present. While no virus was detected in pre-basic mother plants of *Fragaria* and *Ribes* species, rubus yellow net virus (RYNV) was detected in pre-basic mother plants of *Rubus*. Raspberry bushy dwarf virus (RBDV), black raspberry necrosis virus (BRNV), raspberry vein chlorosis virus (RVCV) and RYNV were detected in the *Rubus* genetic resource collection. The L polymerase encoding sequence characterized from seven RVCV isolates showed considerable genetic variation. The data provide the first molecular biological evidence for the presence of RYNV in Finland. RYNV was not revealed in virus indexing by indicator plants, which suggests that it may be endogenously present in some raspberry cultivars. In addition, a putative new RYNV-like badnavirus was detected in *Rubus* spp. Blackcurrant reversion virus (BRV) and gooseberry vein banding associated virus (GVBaV) were detected in symptomatic *Ribes* plants grown in the field. Results were consistent with those obtained using PCR or reverse transcription PCR and suggest that the current virus indexing methods of pre-basic mother plants work as expected. Furthermore, many new viruses were identified in the collections of plant genetic resources not previously tested for viruses. In the future, siRNA-based diagnostics could be a useful supplement for the currently used virus detection methods in certified plant production and thus rationalize and simplify the current testing system.

## Introduction

Virus-free and genetically true-to-type propagation materials are crucial for certified production of small fruits. Certified production of small fruits has been carried out in Finland since 1977 [1]. Pre-basic mother plants must be virus-free and are tested using recommended indexing procedures [1–5]. Basic methods consist of bioassays such as sap-inoculation and graft-inoculation from the tested plant to recommended indicator plants that display symptoms when infected, and serological assays such as enzyme-linked immunosorbent assays (ELISA). In addition, nucleic acid analysis–based methods, such as polymerase chain reaction (PCR) and reverse transcription (RT)-PCR, have become commonplace. Pathogens not permissible in pre-basic mother plants of small fruits are regulated by a decree announced by the European Union (EU) in 2017. The new legislation will change the testing requirements and requires more frequent testing of pests in many plant species.

The national plant genetic resources program of agriculture, horticulture and forestry was established in Finland in 2003 with the aim to conserve genetic resources and promote their sustainable use. The horticultural plants are conserved in clonal field collections, *in vitro* cultures, and cryopreserved [6, 7]. Because of the short growing season and cold winter, only a limited selection of plant species and cultivars can be maintained outdoors in Finland. On the other hand, in summer the long day length and daily variation in temperature are beneficial for accumulation of vitamins and aromatic substances in small fruits [8]. The most important plant species in 2017 were strawberry [*Fragaria x ananassa* (Weston) Royer] grown on 3800 ha, raspberry on 429 ha, and currants (*Ribes nigrum* L. and *R*. *rubrum* L.) on 1740 ha [9].

The maintenance of vegetatively propagated plants free of pathogens is challenging in the field, because plants such as small fruits tend to become infected with viruses [10]. The raspberry aphid (*Aphis idaei* van der Goot) and the European large raspberry aphid (*Amphorophora idaei* Börner) are the most common vectors that transmit viruses to red raspberry (*Rubus idaeus* L.) in Northern Europe [11–12], whereas various *Cecidophyopsis* mites transmit viruses to *Ribes* species [13]. Strawberry aphid (*Chaetosiphon fragaefoli* Cockerell) transmitting viruses to *Fragaria* species has not yet been detected in Finland but was recently detected in Sweden, a neighboring country [14].

The aim of this study was to test viruses in pre-basic mother plants and conserved accessions of plant genetic resources for the presence of viruses. The plants were tested using a newly developed method, deep sequencing of virus-derived small interfering RNAs (siRNAs) in plants, which relies on the ability of plants to recognize and inactivate double-stranded RNA by splicing it into fragments of certain sizes [15, 16–19]. The method is fast and cost-effective, as it allows detection of unrelated viruses simultaneously. Furthermore, RNA samples can be pooled prior to analysis, enabling the detection of large numbers of viruses from different samples simultaneously.

## Materials and methods

### Plant material

**Fig 1:**
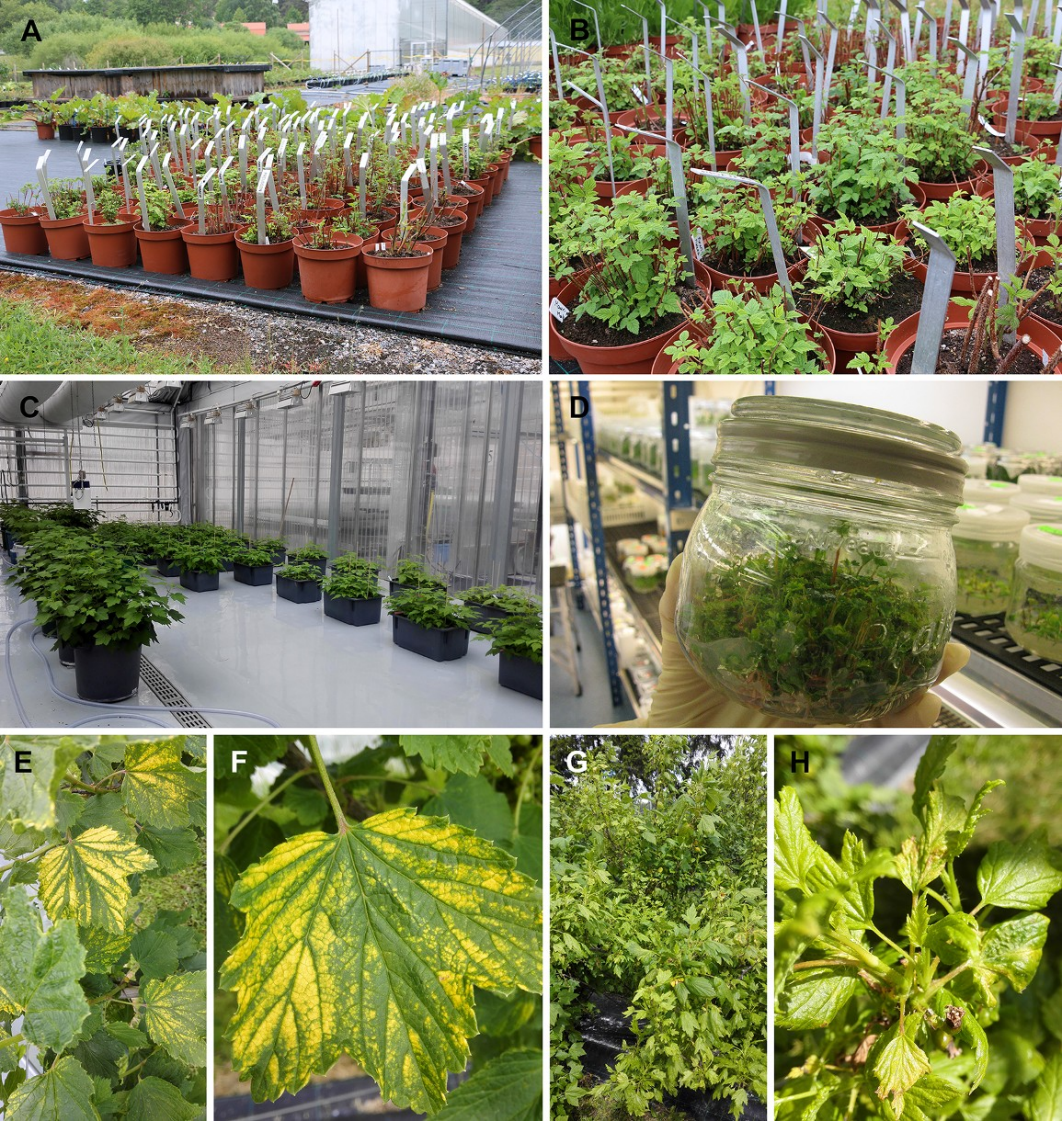
Plant stocks maintained by Luke. (a and b) Outdoor preservation of *Rubus* collection of Finnish plant genetic resources in Piikkiö. (c) Pre-basic mother plants of *Ribes* (right) and *Rubus* (left) in a greenhouse. (d) Pre-basic mother plants of *Fragaria* maintained *in vitro*. (e and f) Leaves of *Ribes rubrum* showing conspicuous yellowing. (g and h) *Ribes nigrum* displaying virus-like malformed leaves in the field.

Pre-basic mother plants of economically important cultivars and other cultivars of *Fragaria*, *Rubus* and *Ribes* spp. suitable for Finnish climate were maintained at the Natural Resources Institute Finland (Luke) in Laukaa (Luke-Laukaa, 62°33’N, 25°99’E) (Fig 1). The plants had been tested for viruses at the intervals stipulated in the rules for maintenance using the recommended methods (ELISA, PCR, graft inoculation and sap inoculation to herbaceous hosts) [3–5] and were maintained in an insect-proof greenhouse (Fig 1C). Also, a few samples of *Rubus* breeding lines (not yet approved as pre-basic mother plants) and indicator plants of *Fragaria* and *Rubus* maintained *in vitro* were tested (Fig 1D, S1 Table). Each propagation line originating from a single meristem was tested individually. For each cultivar/line, there were 1–7 propagation lines. In 2013, several leaves were taken from each pre-basic mother plant of *Ribes* and *Rubus* to obtain representative leaf samples of each propagation line. Samples were stored at −80°C at Luke in Jokioinen (Luke-Jokioinen, 220 km southwest of Laukaa). In addition, similar representative samples were taken from the pre-basic mother plants of *Fragaria* and from the *Rubus* stock plants used as virus indicators and maintained *in vitro*.

The Finnish plant genetic resources collection of *Rubus* was developed in the 1980s. Accessions in the collection originate from different parts of Finland and consist of material selected based on interesting breeding traits, such as winter hardiness. After several rounds of selection, the *Rubus* collection currently consists mainly of *Rubus* cultivars bred in Finland. They are maintained in pots in the field during the summer and in cold storage during the winter at Luke in Piikkiö by the Baltic Sea (Luke-Piikkiö; 350 km southwest from Luke-Laukaa). Plants of the *Rubus* collection had not been previously tested for viruses. The plants were sampled at Luke-Piikkiö in June 2015 (S1 Table), except *Rubus idaeus* cv. Indian Summer, which was maintained and sampled at Luke-Mikkeli (330 km northeast from Luke-Piikkiö). Samples were collected from one to three plants and parallel samples were taken from each cultivar or plant stock. Several leaves, mostly those showing symptoms, were sampled from each plant. In addition, samples were collected from *Ribes nigrum* and *Ribes rubrum* that showed distinct symptoms of malformed leaves and conspicuous yellowing, respectively (Fig 1E–1H). These *Ribes* plants were from a field trial at Luke-Piikkiö and were not included in the germplasm collection (S1 Table). All samples were stored at −80°C.

### RNA isolation, siRNA sequencing and deposition of raw siRNA reads

Total RNA was extracted from leaves as described [20–21] with some modifications to diminish interference of secondary metabolites, phenolics, and polysaccharides, especially for the *R*. *rubrum* samples. RNA concentration and purity were determined with Gene Quant or Nanodrop 2000c UV-vis Spectrophotometer (Thermo Scientific, Wilmington, DE, USA). Equal amounts of total RNA (100 ng) were combined from each leaf sample to obtain six pools, each of which contained 21–43 samples (S1 Table). Four pools (GEN17–20) contained samples from the pre-basic mother plants of certified plant production, including *Fragaria* (GEN17), *Fragaria* and *Rubus* (GEN18), *Rubus* and *Ribes* (GEN19), and *Ribes* (GEN20) (S1 Table). Samples in two pools (HXR1 and HXR2) were from the plant genetic resource collection (S1 Table). Pool HXR1 contained only *Rubus* samples, whereas HXR2 also contained two samples from symptomatic *Ribes* plants grown in the field.

The RNA pools were sent to Fasteris SA (Plan-les-Outes, Switzerland) for sequencing of the small RNAs. RNA samples were subjected to acrylamide gel electrophoresis in Fasteris, and small RNAs < 45 nt long were purified from the gel. Single-stranded 3’ adapters and barcoded 5’ adapters were ligated to the small-RNA oligonucleotides, followed by reverse-transcription and amplification by PCR to generate DNA colony template libraries. The PCR products were purified and diluted to 10 nM prior to high-throughput DNA sequencing by Illumina Genome Analyzer (HiSeq 2500). The raw siRNA reads obtained by Illumina were deposited to the European Nucleotide Archive (ENA) with accession numbers ERP108051 (gene pools GEN19-GEN20) and PRJEB30660 (gene pools HXR1 and HXR2).

### DNA isolation and cDNA synthesis used for PCR analysis

For PCR analysis of DNA viruses, DNA was extracted from fresh or frozen leaves using DNeasy Plant Mini Kit (Qiagen, Hilden, Germany), the cetyl trimethyl ammonium bromide (CTAB) method, or a combination of organic extraction and the CTAB method [22], http://cshprotocols.cshlp.org/content/2010/11/pdb.prot5515. For PCR analysis of RNA viruses, RNA was extracted from frozen leaves with the RNeasy Plant Mini Kit (Qiagen). Total RNA was treated with DNase I (Thermo Scientific) and cDNA was synthesized using the Revert Aid RT Kit (Thermo Scientific) and random hexamer primers. The quality of the cDNA was checked in control PCR reactions using the Phire Plant Direct PCR Kit (Thermo Scientific).

### PCR analysis of samples

For PCR analysis, cDNA was diluted 10-fold to minimize the concentration of possible compounds that may inhibit PCR reactions. PCR was carried out using puReTaq Ready-To-Go PCR Beads (GE Healthcare UK Limited), or Phusion DNA polymerase or PCR Phire Hot Start II DNA polymerase (Thermo Scientific, Vilnius, Lithuania), and virus-specific primers (S2 Table). PCR products were purified using the E.Z.N.A. Gel Purification Kit (Omega BioTech Inc., Norcross, GA, USA) or QIAquick Gel Extraction Kit (Qiagen). They were sequenced by Macrogen (The Netherlands) or in the sequencing facility at Luke-Jokioinen. Most samples were sequenced directly without cloning, but a few PCR products were cloned into the vector pJET (Thermo Scientific) for sequencing using standard methods.

### Data analysis

The reads of 21–24 nt from high-throughput DNA sequencing data were assembled into contigs using Velvet software [23]. The contigs were then used in database searches to find homologous sequences and identify viruses present in the sample pools. Mapping of siRNA reads to the virus sequences identified by BLAST (E-value threshold of 1.00E-10) was carried out with Bowtie [24] in order to examine the coverage and depth of siRNA reads in virus sequences. Analyses were carried out in parallel using the VirusDetect pipeline [25] freely available at http://bioinfo.bti.cornell.edu/tool/VirusDetect/.

Nucleotide and amino acid sequences were aligned using MultAlign [26]. Phylogenetic relationships were analyzed using the neighbor-joining method [27] implemented in MEGA7 [28] using the Kimura two-parameter model [29] for nucleotides and the Poisson model for amino acid sequences. Statistical significance of tree branching was tested by performing 1000 bootstrap replications.

## Results

### Viruses detected by siRNA sequencing

Sequencing of the pooled RNA from the plant samples resulted in 10.0–15.9 million reads (21–24 nt) per pool. No virus was detected in sample pools GEN17 and GEN20, which contained samples from pre-basic mother plants of *Fragaria* and *Ribes*, respectively (Table 1, S1 Table).

**Table 1:** Viruses detected in the sample pools of pre-basic mother plants and some breeding lines.

Accession number^1^	Virus	Sequence (length)	Coverage (%)^2^	Average depth^3^
**Sample pool GEN17**				
	No virus detected			
**Sample pool GEN18**				
FR687353.1	RBDV^**4**^	RNA1 (5401 nt)	72.6	27.9
FR687358.1	RBDV	RNA2 (2183 nt)	80.3	19.6
KF241951.1	RYNV^**5**^	DNA (7932 nt)	10.4	31.8
**Sample pool GEN19**				
KF241951.1	RYNV	DNA (7932 nt)	81.8	35.4
**Sample pool GEN20**				
	No virus detected			

^1^ NCBI database: https://www.ncbi.nlm.nih.gov/.

^2^ Coverage (%) of identical 21- to 24-nt siRNAs relative to the full-length viral reference sequence.

^3^ Average number of times the nucleotides in the reference genome were covered by the siRNA reads of the sample (identical nucleotides, no mismatches allowed).

^4^ RBDV, raspberry bushy dwarf virus.

^5^ RYNV, rubus yellow net virus.

Raspberry bushy dwarf virus (RBDV) and raspberry yellow net virus (RYNV) were detected in pool GEN18, whereas only RYNV was detected in pool GEN19 (Table 1). GEN18 contained samples from pre-basic mother plants of *Fragaria*, a few breeding lines of *Rubus*, and a few virus indicator plants of *Fragaria* and *Rubus*. GEN19 contained samples from pre-basic mother plants of *Rubus* and *Ribes*, a few breeding lines of *Rubus*, and virus indicator plants of *Rubus* (S1 Table).

Mapping of the virus-derived siRNA reads to the RBDV RNA1 and RNA2 showed high coverage of 72.6% and 80.3%, respectively, and high depth of coverage of 27.9 and 19.6, respectively (Table 1). High coverage with siRNA reads (81.8%) of the RYNV genome was observed in pool GEN19, whereas only low coverage (10.4%) was observed in pool GEN18 (Table 1).

Pools HXR1 and HXR2 contained samples from the collection of *Rubus* plant genetic resources (S1 Table). Four known raspberry viruses—RBDV, RYNV, black raspberry necrosis virus (BRNV), and raspberry vein chlorosis virus (RVCV)—were detected in pool HXR1 (Table 2). Five viruses—RBDV, RYNV, BRNV, blackcurrant reversion virus (BRV), and gooseberry vein banding associated virus (GVBaV)—that had infected raspberries or *Ribes* plants were identified in the sample pool HXR2 (Table 2), which contained also one symptomatic plant each of *R*. *nigrum* and *R*. *rubrum* grown in the field. Mapping of the virus-derived siRNAs to viral genomes, including to the partial sequence of RVCV, showed high coverage (60.7–89.9%) of all identified viruses, except BRNV, which had a low coverage of 11.6–24.2% (Table 2).

**Table 2:** Viruses detected in the sample pools from the *Rubus* collection of plant genetic resources (pools HXR1-2) and two *Ribes* plants (pool HXR2).

Accession number^1^	Virus^2^	Sequence (length)	Coverage (%)^3^	Average depth^4^
**Sample pool HXR1**				
FR687353.1	RBDV	RNA1 (5401 nt)	79.3	67.2
FR687358.1	RBDV	RNA2 (2183 nt)	87.8	39.5
KF241951.1	RYNV	DNA (7932 nt)	80.7	28.4
HE611022.1	BRNV	RNA1 (7528 nt)	24.2	17.7
HE614901.1	BRNV	RNA2 (6326 nt)	11.6	21.5
FN812699.2	RVCV	partial L gene (3030 nt)	60.7	7.7
**Sample pool HXR2**				
FR687353.1	RBDV	RNA1 (5401 nt)	86.0	101.6
FR687358.1	RBDV	RNA2 (2183 nt)	89.9	79.3
KF241951.1	RYNV	DNA (7932 nt)	87.7	59.2
HE611022.1	BRNV	RNA1 (7528 nt)	16.3	74.7
HE614901.1	BRNV	RNA2 (6326 nt)	16.0	57.8
AF368272.1	BRV	RNA1 (7711 nt)	68.0	30.6
AF020051.3	BRV	RNA2 (6405 nt)	64.3	39.7
HQ852251.1	GVBaV	DNA (7663 nt)	73.2	11.3

^1^ NCBI database: https://www.ncbi.nlm.nih.gov/.

^2^ BRNV, black raspberry necrosis virus; BRV, blackcurrant reversion virus; GVBaV, gooseberry vein banding associated virus; RBDV, raspberry bushy dwarf virus; RVCV, raspberry vein chlorosis virus; RYNV, rubus yellow net virus.

^3^ Coverage of identical 21- to 24-nt siRNAs relative to the full-length viral reference sequence; in case of RVCV, coverage of the RNA-dependent RNA polymerase encoding region (partial L gene).

^4^ Average number of times the nucleotides in the reference genome were covered by the siRNA reads of the sample (identical nucleotides, no mismatches allowed).

The samples included in the four pools tested by VirusDetect (and usingVelvet and BLAST search) that were found to contain viruses were also tested by PCR or RT-PCR to confirm the results. As expected, RYNV and RBDV were detected in pool GEN18 (S1 Table). RYNV was detected in *Rubus* breeding line Z-22, whereas RBDV was identified in *Rubus* breeding line Z-13 (maintained as a positive control for RBDV). RYNV was detected by PCR in 10 samples from pool GEN19 (S1 Table). The RYNV-positive samples were pre-basic mother plants of three Finnish raspberry cultivars (Maurin Makea, Takalan Herkku, and Jatsi), the commonly grown Canadian cultivar Muskoka, and a breeding line of *Rubus* Z-23. RBDV was detected in 14 plants, BRNV in 13 plants, RYNV or a RYNV-like virus in 15 plants, and RVCV in 8 plants in samples from the *Rubus* collection of plant genetic resources (pools HXR1 and HXR2) (Tables 3 and 4). BRV was detected in a sample of *R*. *nigrum*, and GVBaV was detected in a sample of *R*. *rubrum* (Table 4).

**Table 3:** Viruses detected in pool HXR1 consisting of raspberry samples from the *Rubus* collection of plant genetic resources grown in the field in Piikkiö.

Sample	*Rubus* cultivar	Virus^1^			
		RBDV	BRNV	RYNV	RVCV
**1**	Jenkka	+	-	-	+
**2**	Jenkka	-	-	-	-
**3**	Maurin Makea	+	+	+	-
**4**	Maurin Makea	-	+	+	-
**5**	RU20 Preussen	+	-	-	-
**6**	RU20 Preussen	-	-	-	-
**7**	RU53	-	-	-	+
**8**	RU53	-	-	-	+
**9**	RU54	-	-	-	+
**10**	RU54	-	-	-	+
**11**	RU168 Krusenbergs	+	+	(+)^2^	-
**12**	RU168 Krusenbergs	+	+	(+)^2^	-
**13**	RU55	+	-	-	+
**14**	RU55	-	-	-	+
**15**	Pisan Keltainen	-	-	-	-
**16**	Pisan Keltainen	+	-	+	-
**17**	Uusikirkko	+	-	-	-
**18**	Uusikirkko	-	-	-	-
**19**	HY 6230	-	-	-	-
**20**	HY 6230	-	-	(+)^2^	-
**21**	RU25 Norna	+	-	-	+

^1^ +, virus detected: BRNV, black raspberry necrosis virus; RBDV, raspberry bushy dwarf virus; RVCV, raspberry vein chlorosis virus; RYNV, rubus yellow net virus.

^2^ (+), RYNV-like sequence.

**Table 4:** Viruses detected in pool HXR2 consisting of 19 raspberry samples from the *Rubus* collection of plant genetic resources and two *Ribes* samples grown in the field.

Sample	*Rubus*/*Ribes* cultivars	Virus^1^				
		RBDV	BRNV	RYNV	BRV	GVBaV
**22**	RU25 Norna	+	-	+	-	-
**23**	RU158 Hoolin kanta	-	+	+	-	-
**24**	RU158 Hoolin kanta	-	+	+	-	-
**25**	R159 Ranta, Kaukonen	+	+	-	-	-
**26**	R159 Ranta, Kaukonen	+	+	-	-	-
**27**	RU24, Heija	-	+	-	-	-
**28**	RU24, Heija	-	+	+	-	-
**29**	R156 Ojanperä, Kaukonen	-	+	-	-	-
**30**	R156 Ojanperä, Kaukonen	-	-	-	-	-
**31**	Majestät	-	-	-	-	-
**32**	Majestät	-	-	-	-	-
**33**	RU18 Heisa	-	+	+	-	-
**34**	RU18 Heisa	-	+	+	-	-
**35**	HY 71029	-	-	-	-	-
**36**	HY 71029	-	-	-	-	-
**37**	Ville	+	-	+	-	-
**38**	Ville	+	-	-	-	-
**39**	Indian Summer	-	-	+	-	-
**40**	Indian Summer	-	-	+	-	-
**41**	*Ribes nigrum* (Mara)	-	-	-	+	-
**42**	*Ribes rubrum* (Valkoinen Suomalainen)	-	-	-	-	+

^1^ BRNV, black raspberry necrosis virus; BRV, blackcurrant reversion virus; GVBaV, gooseberry vein banding associated virus; RBDV, raspberry bushy dwarf virus; RYNV, rubus yellow net virus.

### Sequence variability of RYNV

The sequence variability of RYNV was further characterized because only a partial sequence of the open reading frame 3 (ORF3) (AF468454, [29]) and two full-length sequences of RYNV, namely RYNV-Ca (accession number KF241951, [31]) and RYNV-BS (KM078034, [32]), are known and available in GenBank. In contrast, RBDV and BRNV sequences were not further characterized here, as their sequence variability has been relatively well studied [21, 33–35].

A small genomic region (559–844 nt) of ORF3 of RYNV was amplified and sequenced from 22 RYNV-positive *Rubus* samples from pre-basic mother plants and from plants in the collection of genetic resources (Tables 3 and 4). The sequences of RYNV-Ca and RYNV-BS, as well as the variant of the latter (RYNV-BSa; AF468454) were included for comparison (S1 Fig). RYNV-Ca originates in a symptomatic red raspberry in Canada, whereas RYNV-BS has been described from red raspberry cv. “Baumforth’s Seedling A”, which originates in the United Kingdom and has been maintained in Canada. All Finnish RYNV isolates sequenced in this study showed higher sequence identity to RYNV-Ca than RYNV-BS. RYNV isolates from Finnish raspberry cultivars Maurin Makea and Takalan Herkku from pre-basic mother plants of *Rubus* (isolates MM-78, MM-79, MM-80, and TH-88), the *Rubus* collection of plant genetic resources (isolates MM-3dg and MM-4), and also the RYNV isolate PK-16 from raspberry cultivar Pisan keltainen (*Rubus idaeus* f. *chlorocarpus* Krause) were identical (Table 5). The two sequences from raspberry cultivar Muskoka (Mu-81 and Mu-83) and *Rubus* breeding line Z23 (Z23-97 and Z23-98) were identical and also were very similar to each other, with substitutions at only three nucleotide positions. Fourteen of the 22 RYNV isolates sequenced in this study differed at the nucleotide level (S1 Fig). Sequencing of a few isolates resulted in double peaks (degenerate nucleotides) that were detected by direct sequencing of the PCR products (S1 Fig).

**Table 5:** The genomic regions and accession numbers of the RYNV, RYNV-like and RVCV isolates from Finland sequenced in this study, and the reference sequences of RYNV and RVCV isolates included for comparison. Identical sequences obtained from different plants are marked with the same letter in the last column, whereas unique sequences are marked with asterisk (*).

Pools of samples	Sample number^a^	Virus	Isolate	Genomic region	Accession number^b^	Identical sequences
GEN-19	51	RYNV	Jatsi-109	partial ORF3	MH423497^f^	*
	52	RYNV	MM-78	partial ORF3	MH423490^c^	A
	53	RYNV	MM-79	partial ORF3	MH423491^c^	A
	54	RYNV	MM-80	partial ORF3	MH423492^c^	A
	55	RYNV	Mu-81	partial ORF3	MH423493^g^	B
	57	RYNV	Mu-83	partial ORF3	MH423494^g^	B
	61	RYNV	TH-87	partial ORF3	MH423495^c^	*
	62	RYNV	TH-88	partial ORF3	MH423496^c^	A
	70	RYNV	Z23-97	partial ORF3	MH423488^c^	C
	71	RYNV	Z23-98	partial ORF3	MH423489^c^	C
HXR-1	3	RYNV	MM-3dg	partial ORF3	MH347356^d^	D
	3	RYNV	MM-3	partial ORF3, ORF4, ORF5 and ORF7	MH347357^e^	*
	4	RYNV	MM-4	partial ORF3	MH347356^d^	D
	11	RYNV-like	Krus-11	partial ORF3	MH427643^d^	E
	12	RYNV-like	Krus-12	partial ORF3	MH427643^d^	E
	16	RYNV	PK-16	partial ORF3	MH347356^d^	D
	20	RYNV-like	HY-20	partial ORF3	MH427643^d^	E
	8	RVCV	RVCV-8	partial L polymerase	MH388763^i^	F
	9	RVCV	RVCV-9-3	partial L polymerase	MH388763^i^	F
	9	RVCV	RVCV-9-4	partial L polymerase	MH388761^i^	G
	10	RVCV	RVCV-10	partial L polymerase	MH388763^i^	F
	13	RVCV	RVCV-13	partial L polymerase	MH388761^i^	G
	14	RVCV	RVCV-14	partial L polymerase	MH388761^i^	G
	21	RVCV	RVCV-21	partial L polymerase	MH388762^i^	*
HXR-2	22	RYNV	Norna-22	partial ORF3	MH347347^h^	*
	23	RYNV	HK-23	partial ORF3	MH347348^h^	*
	24	RYNV	HK-24	partial ORF3	MH347349^h^	*
	28	RYNV	Heija-28	partial ORF3	MH347350^h^	*
	33	RYNV	Heisa-33	partial ORF3	MH347351^h^	*
	34	RYNV	Heisa-34	partial ORF3	MH347352^h^	*
	37	RYNV	Ville-37	partial ORF3	MH347353^h^	*
	39	RYNV	IS-39	partial ORF3	MH347354^h^	*
	40	RYNV	IS-40	partial ORF3	MH347355^h^	*
Reference sequences:						
		RYNV	RYNV-Ca^b^	full genome	KF241951	[31]
		RYNV	RYNV_BSa^b^	partial ORF3	AF468454	[30]
		RYNV	RYNV-BS^b^	full genome	KM078034	[32]
		RVCV	RVCV^b^	L polymerase gene	FN812699	[34]

^a^ Number of the sample (plant) tested. For further information, see S1 Table).

^b^ NCBI accession numbers of the sequences analyzed in this study. The previously analyzed four reference sequences are at the end of the table.

^c^ The partially sequenced ORF3 (844 nt) corresponds to nucleotides 6036–6879 of full length RYNV-Ca (14.3% of ORF3).

^d^ The partially sequenced ORF3 (559 nt) corresponds to nucleotides 6282–6840 of full length RYNV-Ca (9.4% of ORF3). The sequence is identical to the corresponding region in isolates marked with ‘A’ (MM-78, MM-79, MM-80 and TH-88).

^e^ ORF4, ORF5 and ORF7, and the partially sequenced ORF3 correspond to nucleotides 6081–7932 and 1–496 of RYNV-Ca (KF241951), respectively.

^f^ The partially sequenced ORF3 (590 nt) corresponds to nucleotides 6254–6843 of RYNV-Ca (10.0% of ORF3).

^g^ The partially sequenced ORF3 is 559 (Mu-81) and 598 (Mu-83) nucleotides and corresponds to nucleotides 6282–6840 and 6282–6879, respectively, of RYNV-Ca (9.4% and 10.1% of ORF3, respectively). The sequences are identical in the 559-nt region common to both sequences.

^h^ The partially sequenced ORF3 (577 nt) corresponds to nucleotides 6282–6858 of full length RYNV-Ca (9.8% of ORF3).

^i^ The sequenced region of Finnish RVCV isolates was 827 nt and. It corresponds to nt 265–1091 of the RVCV isolate FN812699 (27.3% of the L polymerase encoding region).

The internal nucleotide sequence variability was identical or very similar in samples that were derived from the same raspberry cultivars, especially in the samples from pre-basic mother plants of *Rubus*. They included MM-78, MM-79, and MM-80 (raspberry cultivar Maurin Makea); TH-87 and TH-88 (raspberry cultivar Takalan Herkku); and Jatsi-109 (raspberry cultivar Jatsi) in pre-basic mother plants of raspberry and also in raspberry isolates MM-3dg, MM-4 (cultivar Maurin Makea), and PK-16 (cultivar Pisan keltainen) in the *Rubus* collection of genetic resources (S1 Table, S1 Fig). In contrast, no degenerate nucleotides were detected in isolates Z23-97 and Z23-98 (raspberry line Z-23) or in Mu-81 and Mu-82 (cultivar Muskoka) from pre-basic mother plants of *Rubus*, nor in isolates Norna-22 (cultivar RU25 Norna), HK-23 and HK-24 (cultivar RU158 Hoolin kanta), Heija-28 (cultivar RU24 Heija), Heisa-33 and Heisa-34 (from cultivar RU18 Heisa), Ville-37 (from cultivar Ville), or IS-39 and IS-40 (from cultivar Indian Summer) from the *Rubus* collection of genetic resources (S1 Table, S1 Fig). In general, RYNV sequences amplified from the *Rubus* collection of plant genetic resources grown in the field showed more variability than the isolates from pre-basic mother plants.

**Fig 2:**
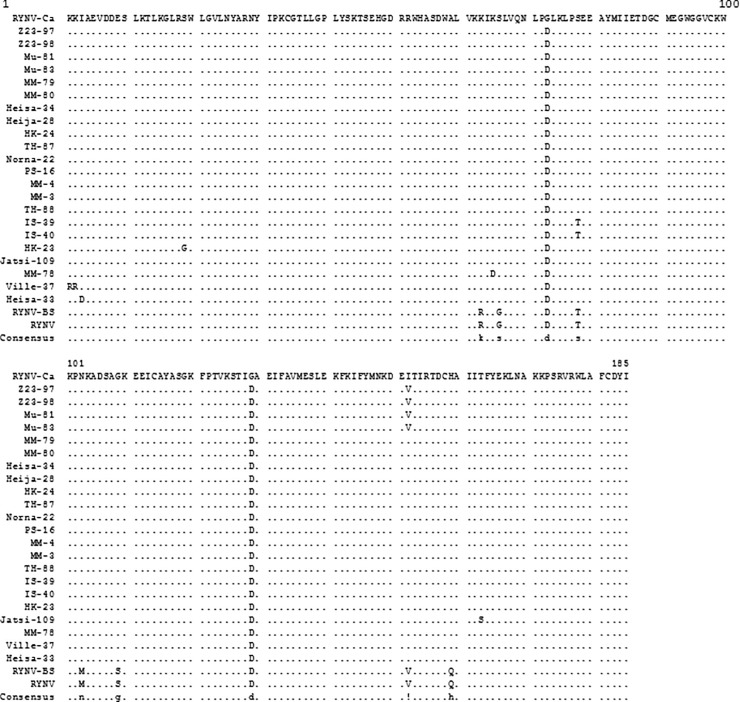
Multiple alignment of the deduced amino acid sequences of the open reading frame 3 (ORF3) of rubus yellow net virus (RYNV). ORF3 of 22 RYNV isolates were sequenced and the deduced amino acid sequences corresponding to amino acids 1618–1802 of ORF3 in RYNV-Ca were compared with the sequences in RYNV-Ca (KF241951), RYNV-BS (KM078034) and RYNV-BSa (AF468454). Only the amino acids that differ from those of RYNV-Ca are shown. Identical amino acids are indicated by dots. The degenerate nucleotides in various positions (see S1 Fig) did not result in amino acid substitutions.

Comparison of the deduced amino acid sequences indicated that all Finnish RYNV isolates were very similar (97.3–100% aa identity) and nearly identical to RYNV-Ca (96.8–98.9% aa identity) (Fig 2) over the part of the viral genome analyzed. In addition, the degenerate nucleotides detected in the sequences were synonymous at amino acid level, and only 9 RYNV isolates out of 22 differed at the amino acid level.

Sequence variability of RYNV was further characterized by cloning and sequencing PCR products of RYNV isolates from raspberry cultivar Maurin Makea. Analysis of 10 PCR clones indicated variability at several nucleotide positions. Most of this variability occurred at the earlier-detected degenerate nucleotide positions rather than at unique positions (S2 Fig). Five of the 10 analyzed RYNV clones differed in their amino acid sequences (S3 Fig).

**Fig 3:**
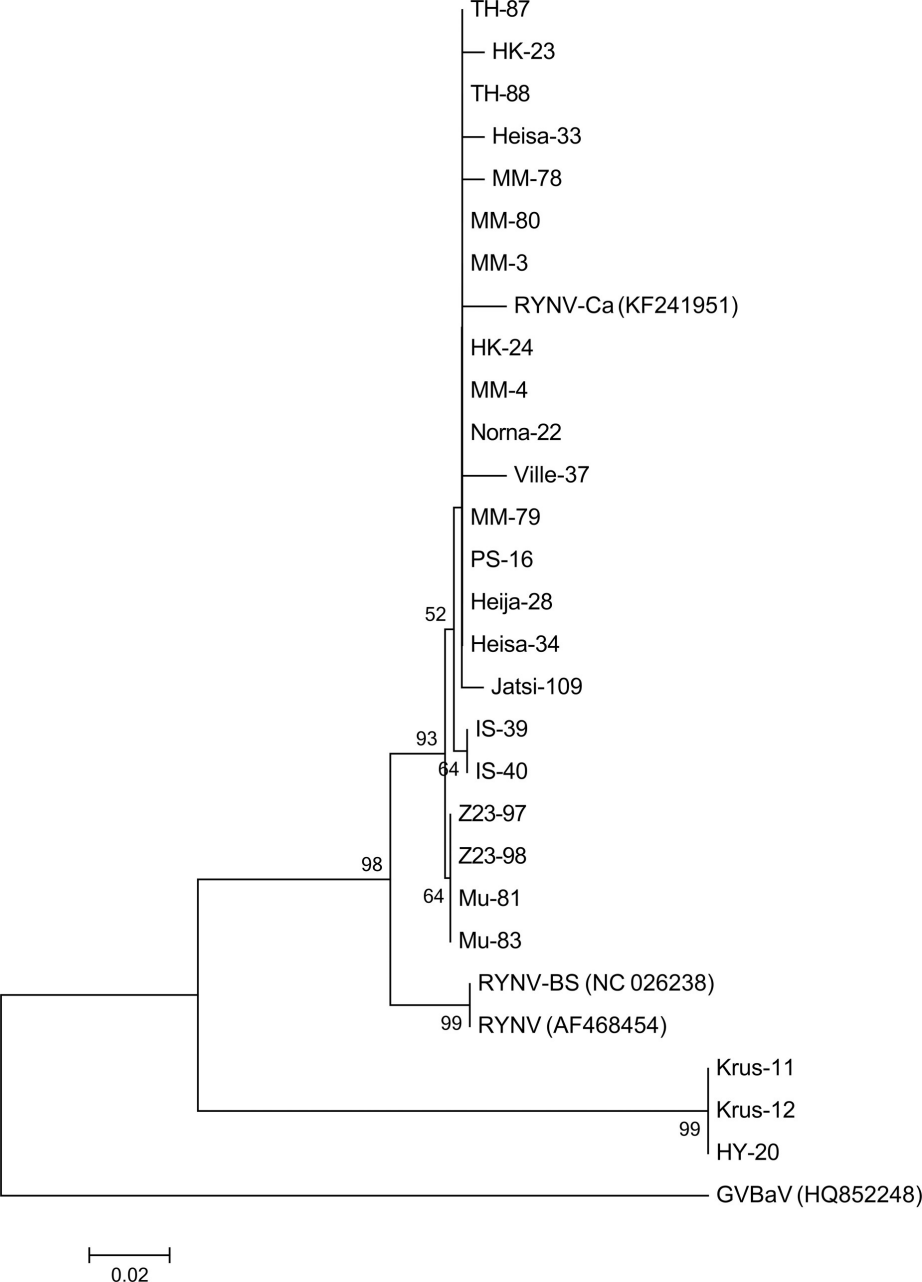
Phylogenetic comparison of deduced partial ORF3 amino acid sequences of 22 Finnish RYNV isolates, three RYNV-like isolates, and two previously described RYNV isolates RYNV-Ca and RYNV-BS from Canada and United Kingdom, respectively, using the neighbor-joining algorithm [26]. GVBaV (HQ852248) was included as a root. Numbers at branches represent bootstrap values of 1000 replicates.

The previously characterized isolates RYNV-Ca and RYNV-BS differ in their genomic organization [32]. To characterize the genomic organization of the Finnish RYNV isolates, a larger genomic region (2348 nt) of isolate MM-3 was amplified and sequenced (see nucleotide alignment in S4 Fig). Genomic organization of MM-3 was similar to that of RYNV-Ca, and the nucleotide sequence of MM-3 was 98.7% identical to that of RYNV-Ca. MM-3 expresses ORF5 from the sense strand and ORF7 from the antisense strand, which is similar to RYNV-Ca. These ORFs were missing from RYNV-BS [32]. ORF5 and ORF7 differed only at four nucleotide positions and one nucleotide position, respectively, which was predicted to cause no change or changes in two amino acids, respectively, between MM-3 and RYNV-Ca. ORF4 was identical among the isolates (S4 Fig). Phylogenetically accessed, all Finnish RYNV isolates grouped to the same large cluster together with RYNV-Ca, whereas RYNV-BS was placed on a separate branch. Finnish isolates were further divided into two subclusters containing the isolates Z23-97, Z23-98, Mu-81, and Mu-83 and the isolates IS-39 and IS-40 (Fig 3).

In addition to isolates of RYNV, a few sequences resembling RYNV were detected in three samples from raspberry cultivars RU168 Krusenbergs (isolates Krus-11 and Krus-12) and HY 6230 (isolate HY-20) (Tables 3 and 5). These three identical isolates were only 71.6% and 83.4% identical to RYNV-Ca and RYNV-BS, respectively, at the nucleotide level, and 75.6% and 85.6% identical, respectively, at the deduced amino acid level **(**S5 Fig**)**. In phylogenetic analysis, the three identical RYNV-like isolates formed a clearly distinct clade supported by a high (99%) bootstrap value **(**Fig 3**)**. Hence, they may represent a new badnavirus species or, alternatively, be remnants of an endogenous virus sequence.

### Sequence variability of RVCV

**Fig 4:**
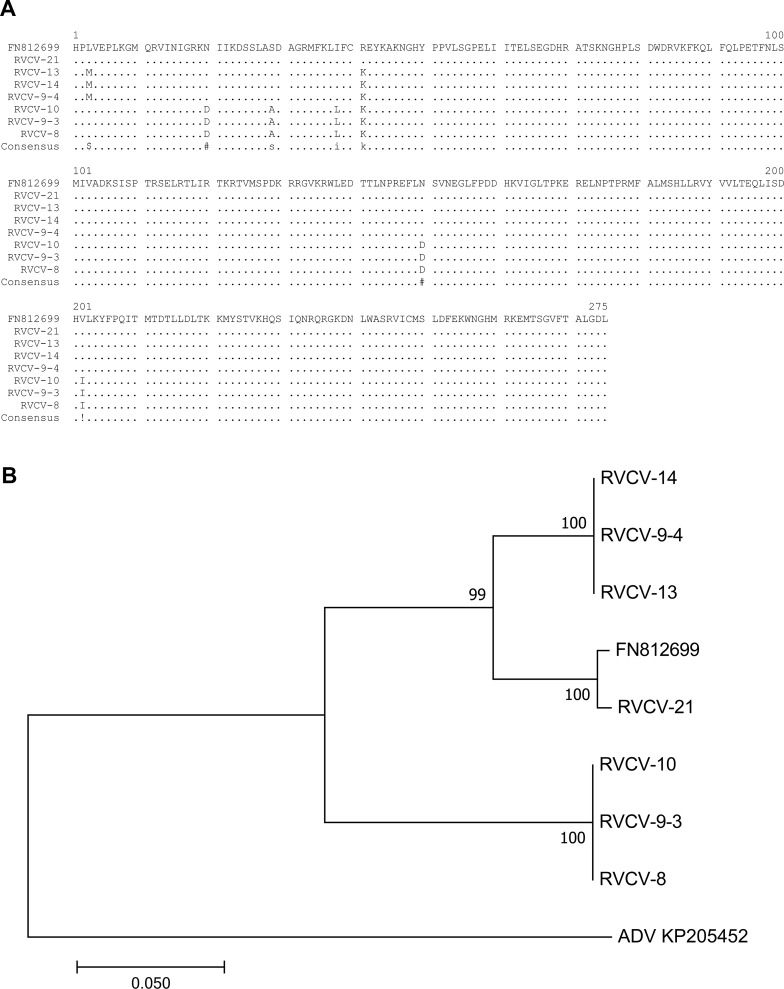
Sequence variability of raspberry vein chlorosis virus (RVCV). (a) Multiple alignment of the amino acid sequences of a fragment of the L polymerase from seven RVCV isolates sequenced in this study and the previously published RVCV sequence (database number FN812699) from Scotland. Amino acids that differ from those of FN812699 are marked by alphabets, and identical amino acids are indicated by dots. The aligned region corresponds to amino acids 89–363 of FN812699. (b) Phylogenetic comparison of the L polymerase encoding region (827 nucleotides) of seven Finnish RVCV isolates and the previously described RVCV (FN812699). Alfalfa dwarf virus (KP205452) was included to root the phylogenetic tree. Numbers at branches represent bootstrap values of 1000 replicates. Isolates RVCV-14, RVCV-9-4 and RVCV-13, as well as isolates RVCV-10, RVCV-9-3 and RVCV-8, were identical.

The only published data on RVCV sequences is a 3030-nt-long region of the L polymerase gene [36]. Therefore, part (827 nt) of the L polymerase–encoding sequence was characterized from seven Finnish RVCV isolates (Table 5). The sequences showed considerable variability at the nucleotide level and differed also from the previously characterized RVCV isolate. Direct sequencing of PCR products revealed double peaks, indicating the presence of different RVCV isolates in individual raspberry plants in the *Rubus* collection of genetic resources in the field. For example, two or more RVCV variants were found in raspberry cultivars RU53 and RU54. Therefore, some of the PCR products were cloned and sequenced, and isolates designated as RVCV-8 (cultivar RU53), RVCV-9-3, RVCV-9-4 and RVCV-10 (Table 3). Identical RVCV isolates, as judged by the sequenced 827-nt long region of the viral genome, were detected in cultivar RU54 and RU55 (Fig 4, Table 5).

Isolate RVCV-21 (cultivar RU25 Norna) was most similar—99.2% at the nucleotide level (S6 Fig) and 100% at the amino acid level (Fig 4A)—to the RVCV isolate from raspberry in Scotland (accession number FN812699). These two isolates formed a subcluster in the phylogenetic tree (Fig 4B). Isolates RVCV-13 and RVCV-14 from raspberry cultivar RU55 and isolate RVCV-9-4 from RU54 were identical and showed 93.1% and 99.3% similarity at the nucleotide and amino acid levels, as compared with the previously identified RVCV (Fig 4A, S6 Fig). They formed a small subcluster in the cluster that contained the previously characterized RVCV and RVCV-21 (Fig 4B). Isolates RVCV-8 from cultivar RU53 and RVCV-9-3 and RVCV-10 from cultivar RU54 were also identical to each other. They also differed most from the Scottish RVCV (84.7% nt and 97.8% aa identity to FN812699) and thus formed a separate small cluster (Fig 4, S6 Fig).

## Discussion

In this study, we tested the presence of viruses in pre-basic mother plants and conserved accessions of plant genetic resourses using the newly developed virus detection method (deep sequencing of virus-derived siRNAs). Four viruses (RBDV, BRNV, RYNV, and RVCV) were detected in the *Rubus* collection of plant genetic resources. These plants have not been tested for the presence of viruses before. In addition, two viruses (BRV and GVBaV) were identified in symptomatic *Ribes* plants grown in a field trial.

Only one virus (RYNV) was detected in pre-basic mother plants of *Rubus* by siRNA-based diagnostics, and no virus was detected in pre-basic mother plants of *Fragaria* and *Ribes* species. The results confirm that thermotherapy combined with meristem tip culture and regular virus indexing generally produce virus-free plants [37]. RYNV and other viruses belonging to the raspberry mosaic virus complex have been tested previously by grafting using *Rubus idaeus* ‘Malling Landmark’, *R*. *idaeus* ‘Malling Delight’ and *Rubus occidentalis* ‘Cumberland’ as indicator plants. However, the results of this study indicate that graft inoculation to indicator plants may be insufficient for indexing method to detect RYNV. RYNV (genus *Badnavirus*, family Caulimoviridae) is one of the viruses associated with raspberry mosaic disease complex, together with BRNV (unassigned genus, family Secoviridae) and raspberry leaf mottle virus (RLMV, genus *Closterovirus*, family Closteroviridae) [10]. Both RYNV and BRNV were detected in this study, whereas no RLMV was found. BRNV is an aphid-transmitted bipartite RNA virus that is present in very low titers in raspberries [38]. Sequences of BRNV isolates from the United Kingdom, the United States, and Finland show large genetic variation [21, 33, 34]. Mixed infection of different *Rubus* viruses (RYNV, BRNV, RBDV, and RVCV) appeared common among plants from the *Rubus* collection of genetic resources grown in the open field in Finland. Of 40 analyzed plant samples, 17 were found to contain two or three viruses. RBDV and BRNV have been previously identified in both cultivated and wild raspberries in Finland [7, 21, 39, 40]. In addition, RBDV has been detected in arctic brambles (*Rubus arcticus* L.) in Finland [39, 41]. RBDV is a pollen-transmitted virus with a bipartite RNA genome [10]. It has a wide host range and worldwide distribution, and its genomic sequence has been well characterized [35, 42–45].

RYNV was not revealed in virus indexing by indicator plants in pre-basic mother plants of *Rubus*, which suggests that it may actually be an endogenous virus that is present in some raspberry cultivars, as suggested [46, 47]. In plants of *Rubus* collection of plant genetic resources, RYNV sequences were variable. All isolates, except the one from Maurin Makea, were unique for their sequence. Some samples also contained sequences that resembled RYNV but were, however, notably different from them. The nucleotide sequence identity of the sequenced ORF3 region of these RYNV-like isolates was <84% as compared with previously characterized RYNV isolates. Thus, they may form a new virus species in the genus *Badnavirus*. Alternatively, they may represent sequences of endogenous badnaviruses that have integrated into the plant genome.

The new RYNV-like virus and RYNV detected in this study were the most common partners in mixed virus infections, followed by RBDV (genus *Idaeovirus*). RBDV in combination with RYNV or other aphid-transmitted viruses, such as RLMV and raspberry latent virus (RpLV), cause severe crumbly fruit symptoms in raspberries [10]. Symptoms of raspberry yellows and yellow mosaic disease and of raspberry vein chlorosis have been described in Finnish raspberry cultivars [48] that could correspond to RYNV and RVCV, respectively. So far, however, no definitive reports or sequence information of these viruses have been available from Finland before this study.

The host range of RYNV is restricted to *Rubus* species. This virus is transmitted by aphid species *Amphorophora agathonica* Hottes in North America and *A*. *idaei* Börner in Europe [29]. Infection by RYNV alone results in the development of a netlike chlorosis along the leaf veins in some raspberry cultivars, but in most raspberry cultivars it is asymptomatic [49]. Jones et al. [29] identified RYNV as a distinct badnavirus. Thus far, two complete sequences of RYNV have been characterized, each of which shows altered genomic organization [31, 32]. The DNA genome of the first sequenced isolate, RYNV-Ca, originated from a red raspberry plant in Canada [50] and consists of 7932 nt and seven ORFs. Five of the ORFs are in a sense orientation and two are on the antisense strand [31]. The isolate RYNV-BS from red raspberry cultivar Baumforth’s Seedling A, originally from England, is 7836 nt long and shows 82% nucleotide identity to isolate Ca. It encodes, however, only five ORFs, of which four are on the sense strand and one is on the antisense strand [32].

This study shows the first molecular biological evidence for the presence of RYNV in Finland. We analyzed a 559-nt region of the conserved ORF3 of RYNV from 22 *Rubus* samples. The samples represented different red raspberry cultivars and breeding lines grown in Finland. All identified Finnish RYNV isolates showed higher sequence similarity to RYNV-Ca, including identical genomic organization, as compared with RYNV-BS. Thus, the data do not support the previously suggested assumption that RYNV-Ca represents a North American lineage and RYNV-BS a European lineage of RYNV [32]. The sequence variation among Finnish RYNV isolates was also relatively large and these isolates were further divided into three subclusters, as the RYNV isolates from cultivars Z23 and Muskoka and those isolates from the cultivar Indian Summer formed two smaller subclusters.

The Finnish RYNV isolates from raspberry cultivars Maurin Makea, Takalan Herkku, and Pisan Keltainen in the largest subcluster contained several variable degenerate nucleotides but, nevertheless, were identical at the amino acid level. Two of these isolates are from the pre-basic mother plants of *Rubus* and represent two Finnish cultivars (Maurin Makea and Takalan Herkku). The cultivars are progeny from crossings by free pollination between wild and cultivated *Rubus* species. The original mother plants show no viral symptoms themselves, and virus-indexing using indicator plants has not revealed any virus. There is increasing evidence that endogenous forms of RYNV exist in some raspberry genomes [46, 47]. Therefore, these isolates may represent an endogenous form of RYNV instead of actively replicating exogenous viruses, as suggested by the data above. The integration of endogenous badnaviruses is assumed to have taken place by illegitimate recombination into host genomes, and their presence is not necessarily associated with infection [51–53].

RVCV belongs to family *Rhabdoviridae*, in which the viruses have a negative-sense RNA genome of 12000–14500 nt, and is common in Europe [10]. Based on symptoms typical to RVCV, it has been reported that RVCV may be common in wild raspberries and the raspberry cultivars Asker, Malling Promise, and Preussen in Finland [48]. Our study provides the first molecular evidence of the presence of RVCV in cultivated raspberries in Finland. RVCV is transmitted by raspberry aphid (*Aphis idaei*) and may easily spread between plants. Symptoms of RVCV include chlorosis of minor veins and reductions in plant vigor and raspberry yields, especially in mixed infections [10]. There is only one published partial RVCV sequence available. It is from the L polymerase–encoding region of the virus [36]. Therefore, no information about genetic variability of RVCV is available. In this study, we show that there is substantial sequence variation among Finnish RVCV isolates, and many plants are simultaneously infected by two or more different RVCV isolates. The characterized Finnish RVCV isolates were detected in a representative collection of cultivated raspberries obtained from different parts of Finland and maintained in Luke-Piikkiö in the field. The nucleotide sequence identity of individually characterized Finnish RVCV isolates, as compared with the previously characterized RVCV isolate, was 93.1–99.2%. However, actual variability is probably higher, as indicated by the numerous degenerate nucleotides in plants infected with a mixture of RVCV isolates.

Symptomatic *Ribes* plants in a field trial in Piikkiö were shown to contain BRV (genus *Nepovirus*) in *R*. *nigrum* and GVBaV (genus *Badnavirus*) in *R*. *rubrum*. BRV is a bi-partite RNA virus that is the causative agent of the reversion disease of blackcurrants. Eriophyid gall mite of blackcurrant (*Cecidophyopsis ribis* Westwood) transmits BRV and also causes substantial damage, including distortion of leaves, galling, and sterility of buds [54]. BRV was originally isolated from reverted blackcurrants and is present in Finland [55, 56]. GVBaV is a DNA virus related to RYNV and is transmitted by aphids. It causes gooseberry vein banding disease in *Ribes* plants and is present in Europe and North America [57].

This study also confirms that siRNA-based diagnostics can be used to detect different viruses in plant samples without previous knowledge about the infecting virus. Recent studies show that VirusDetect reaches a similarly high sensitivity relative to RT-PCR in detection of plant viruses [19]. However, sensitivity might vary depending on the method used for sampling and library preparation, downstream manipulation of RNA and the infection cycles of viruses. That no prior knowledge about viruses that may be present in plant samples is required is an advantage of this method. The method is also relatively fast. This study did raise some questions, such as how to proceed in situations where new, unexpected viruses are detected in the samples. The rules for situations mentioned above are being discussed by the plant inspection authorities [58] who decide the methods used in phytosanitary testing.

In summary, siRNA-based diagnostics revealed only one virus (RYNV) that is putatively endogenous in pre-basic mother plants suggesting that the current virus indexing methods are working as expected. On the other hand, several viruses were detected in accessions of plant genetic resources previously not tested for viruses. This is not unexpected as these plants are maintained in the field. In general, siRNA-based diagnostics proved useful for testing plants for viruses and could be a valuable supplement or replacement of some of the existing methods used for virus detection in pre-basic mother plants for certified plant production, and in the analysis of collections of plant genetic resources.

## Supporting information

S1 Fig Multiple alignment of the nucleotide sequences of the open reading frame 3 (ORF3) genomic region of the 22 rubus yellow net virus (RYNV) isolates sequenced in this study relative to the previously identified sequences of RYNV-Ca (KF241951), RYNV-BS (KM078034) and RYNV (AF468454). Only the nucleotides that differ from those of RYNV-Ca are shown. Identical nucleotides are indicated by dots. The aligned region corresponds to the nucleotides 6282–6840 of the complete nucleotides sequence of RYNV-Ca. Wobble bases: R (A, G), Y (C, T), M (A, C), W (A, T).
(DOCX)

S2 Fig Multiple alignment of nucleotide sequences of the open reading frame 3 (ORF3) genomic region of ten PCR clones of rubus yellow net virus (RYNV) isolate MM-3dg. Each nucleotide sequence indicates sequence from an independent PCR clone of MM-3dg. The uppermost sequence is the sequence obtained by direct sequencing of the PCR product. All sequences are different to each other except the sequences of MM-3-2 and MM-3-3, and MM-3-1 and MM-3-7 are identical to each other.
(DOCX)

S3 Fig Multiple alignment of amino acid sequences of the open reading frame 3 (ORF3) of ten PCR clones of rubus yellow net virus (RYNV) isolate MM-3dg. Each amino acid sequence indicates sequence from an independent PCR clone of MM-3dg. The uppermost sequence is obtained by direct sequencing of the PCR product. X denotes the position containing degenerate nucleotides. The amino acid sequences of the clones 1, 2, 3, 6, 7 and 10 of MM-3dg are identical to each other, whereas the others differ from each other. There were totally five different sequences between the ten sequenced clones.
(DOCX)

S4 Fig Nucleotide alignment and genomic organization of Finnish rubus yellow net virus (RYNV) isolate MM-3 relative to the previously identified sequence of RYNV-Ca (KF241951). Only the nucleotides that differ from those of RYNV-Ca are shown. Identical nucleotides are indicated by dots. The aligned region corresponds to the nucleotides 6081–7920 and nucleotides 1–496 of the complete nucleotide sequence of RYNV-Ca. The open reading frames encoded by the sequences are indicated. 5’-terminal part of ORF1 in green, 3’-terminal part of ORF3 in bold and underlined, ORF4 in blue, ORF5 in red and ORF7 encoded in antisense strand in cursive and underlined.
(DOCX)

S5 Fig Multiple alignment of the open reading frame 3 (ORF3) genomic region of the three rubus yellow net virus (RYNV) like isolates sequenced in this study relative to the previously identified sequences of RYNV-Ca (KF241951) and RYNV-BS (KM078034). **A**, Multiple alignment of nucleotide sequences. Only nucleotides that differ from those of RYNV-Ca are shown. Identical nucleotides are indicated by dots. The aligned region corresponds to the nucleotides 6282–6840 of the complete nucleotides sequence of RYNV-Ca. **B**, Multiple alignment of amino acid sequences. Only amino acids that differ from those of RYNV-Ca are shown. Identical amino acids are indicated by dots.
(DOCX)

S6 Fig Nucleotide sequence alignment of the previously published L polymerase gene fragment of RVCV (database number FN812699) and seven RVCV isolates sequenced in this study. Only the nucleotides that differ from those of FN812699 are shown. Identical nucleotides are indicated by dots. The aligned region corresponds to nucleotides 265–1091 of FN812699. Wobble bases: K (G, T), R (A, G), Y (C, T), M (A, C), S (G, C), W (A, T), N (G, A, T, C).
(DOCX)

S1 Table Plant samples from pre-basic mother plants, breeding lines, and indicator plants from Luke-Laukaa (GEN17 to GEN20), samples from field-grown *Rubus* plants of the genetic resource collection at Luke-Piikkiö and two samples of field grown *Ribes* plants (HXR1 and HXR2). (DOCX)

S2 Table Primers used in the study. (DOCX)
