# Supplementary material for: Small-RNA analysis of pre-basic mother plants and conserved accessions of plant genetic resources for the presence of viruses
Source: PLoS One. 2019 Aug 7;14(8):e0220621. doi: 10.1371/journal.pone.0220621 (PMC6685626; doi:10.1371/journal.pone.0220621)
Supplement: S1 Fig — Only the nucleotides that differ from those of RYNV-Ca are shown. Identical nucleotides are indicated by dots. The aligned region corresponds to the nucleotides 6282–6840 of the complete nucleotides sequence of RYNV-Ca. Wobble bases: R (A, G), Y (C, T), M (A, C), W (A, T). (DOCX) [file pone.0220621.s001.docx]

**S1 Figure. Multiple alignment of the nucleotide sequences of the open reading frame 3 (ORF3) genomic region of the 22 rubus yellow net virus (RYNV) isolates sequenced in this study relative to the previously identified sequences of RYNV-Ca (KF241951), RYNV-BS (KM078034) and RYNV (AF468454).** Only the nucleotides that differ from those of RYNV-Ca are shown. Identical nucleotides are indicated by dots. The aligned region corresponds to the nucleotides 6282-6840 of the complete nucleotides sequence of RYNV-Ca. Wobble bases: R (A, G), Y (C, T), M (A, C), W (A, T).

1 100

RYNV-Ca TCAAGAAGAT AGCTGAGGTG GACGATGAAT CTCTGAAGAC CCTCAAGGGG CTGAGAAGTT GGTTGGGAGT TCTCAACTAT GCCAGGAACT ACATCCCGAA

MM-79 .......... .......... .......... ....R..R.. ......R... T......... .......... Y......... .....R.... ....Y.....

MM-78 .......... .......... .......... ....R..R.. ......R... T......... .......... Y......... .....R.... ....Y.....

PK-16 .......... .......... .......... ....R..R.. ......R... T......... .......... Y......... .....R.... ....Y.....

MM-80 .......... .......... .......... ....R..R.. ......R... T......... .......... Y......... .....R.... ....Y.....

MM-3dg .......... .......... .......... ....R..R.. ......R... T......... .......... Y......... .....R.... ....Y.....

TH-88 .......... .......... .......... ....R..R.. ......R... T......... .......... Y......... .....R.... ....Y.....

MM-4 .......... .......... .......... ....R..R.. ......R... T......... .......... Y......... .....R.... ....Y.....

TH-87 .......... .......... .......... ....R..R.. ......R... T......... .......... Y......... .......... ....Y.....

Jatsi-109 .......... .......... .......... ....R..R.. ......A... T......... .......... Y......... .......... ....Y.....

Norna-22 .......... .......... .......... .......... .......... T......... .......... C......... .....A.... ....T.....

Heija-28 .A........ .......... .......... .......... .......... T......... .......... C......... .....A.... ....T.....

HK-23 .A........ .......... .......... .......... .......... T.....G... .......... C......... .....A.... ....T.....

Ville-37 AA.GA.G... .......... .......... .......... .......... T......... .......... C......... .....A.... ....T.....

HK-24 .A........ .......... .......... ....A..A.. ......A... T......... .......... .......... .......... ..........

Heisa-33 .A......GA T......... .......... .......... ......A... T......... .......... .......... .......... ....T.....

Heisa-34 .A........ .......... .......... .......... ......A... T......... .......... .......... .......... ....T.....

IS-39 .A........ .......... .......... .C..A..... ......A... ........C. .......... ...G...... .......... .......C..

IS-40 .A........ .......... .......... .C..A..... ......A... ........C. .......... ...G...... .......... .......C..

Z23-97 .......A.. ...A...... ........G. .A..C..... .........A T....G.... .......... C........C ..G.....T. ....T..A..

Z23-98 .......A.. ...A...... ........G. .A..C..... .........A T....G.... .......... C........C ..G.....T. ....T..A..

Mu-81 .M........ .......... ........G. .A..C..... .........A T....G.... .......... C........C ..G.....T. ....T..A..

Mu-83 .M........ .......... ........G. .A..C..... .........A T....G.... .......... C........C ..G.....T. ....T..A..

RYNV-BS .A........ ...C...... ..T..C.... .C.....A.. ......A... T.AC....C. .......... G........C ..GC.C.... .......A..

RYNV .A........ ...C...... ..T..C.... .C.....A.. ......A... T.AC.T..C. .......... G........C ..GC.C.... .......A..

Consensus .a........ ...t...... ..c..t..a. .......g.. ......a..g t.ga.a..t. .......... ...c.....t ..ca.g..c. ....c.....

101 200

RYNV-Ca GTGCGGAACA CTCCTAGGCC CACTATACAG CAAGACCAGT GAGCATGGAG ACAGAAGGTG GCATGCTTCG GATTGGGCCT TAGTAAAGAA GATCAAGAGC

MM-79 .......... .....R.... .R........ .........Y .......... .......R.. ......W... .......... .......... ......R...

MM-78 .......... .....R.... .R........ .........Y .......... .......R.. ......W... .......... .......... ......R...

PK-16 .......... .....R.... .R........ .........Y .......... .......R.. ......W... .......... .......... ......R...

MM-80 .......... .....R.... .R........ .........Y .......... .......R.. ......W... .......... .......... ......R...

MM-3dg .......... .....R.... .R........ .........Y .......... .......R.. ......W... .......... .......... ......R...

TH-88 .......... .....R.... .R........ .........Y .......... .......R.. ......W... .......... .......... ......R...

MM-4 .......... .....R.... .R........ .........Y .......... .......R.. ......W... .......... .......... ......R...

TH-87 .......... .....R.... .R........ .........Y .......... .......R.. ......W... .......... .......... ......R...

Jatsi-109 .......... .....R.... .R........ .........Y .......... .......R.. ......W... .......... .......... ......R...

Norna-22 .......... .....G.... .G........ .........C .......... .......A.. ......A... .......... .......... ......A...

Heija-28 .......... .....G.... .G........ .........C .......... .......A.. ......A... .......... .......... ......A...

HK-23 .......... .....G.... .G........ .........C .......... .......A.. ......A... .......... .......... ......A...

Ville-37 .......... .....G.... .G........ .........C .......... .......A.. ......A... .......... .......... ......A...

HK-24 .......... .......... .G........ .........C .......... .......A.. ......A... .......... .......... ......A...

Heisa-33 .......... .......... .G........ .........C .......... .......A.. ......A... .......... .......... ......A...

Heisa-34 .......... .......... .G........ .........C .......... .......A.. ......A... .......... .......... ......A...

IS-39 ...T..G... .....T.... ....C..... ......G... .....C..G. .......A.. ......A... .......... ....C..... A.....A...

IS-40 ...T..G... .....T.... ....C..... ......G... .....C..G. .......A.. ......A... .......... ....C..... A.....A...

Z23-97 .........T ..T..C.... .......... ......T... .....C.... ....G..A.. ...C..A..A .......... .G..C..... ......A...

Z23-98 .........T ..T..C.... .......... ......T... .....C.... ....G..A.. ...C..A..A .......... .G..C..... ......A...

Mu-81 .........T ..T..C.... .......... ......T... .....C.... ....G..A.. ...C..A..A .......... .G..C..... ......A...

Mu-83 .........T ..T..C.... .......... ......T... .....C.... ....G..A.. ...C..A..A .......... .G..C..... ......A...

RYNV-BS ...T..C... ..GT...... .......... .........C ........T. .TC.T..... ...C..G..T .......... ....C..A.G A..T...G..

RYNV ...T..C... ..GT...... .......... .........C ........T. .TC.T..... ...C..G..T .......... ....C..A.G A..T...G..

Consensus ...c..a..a ..cc...... .a..a..... ......c... .....t..a. .ca.a..a.. ...t..a..g .......... .a..c..g.a g..c..aa..

201 300

RYNV-Ca CTGGTCCAAA ATCTCCCAGG CCTCAAACTG CCCAGTGAGG AGGCCTATAT GATCATCGAG ACAGATGGTT GTATGGAAGG ATGGGGCGGA GTCTGTAAGT

MM-79 ........R. .Y.......A .......... .......... .......... .........R .......... .......... W......... ..........

MM-78 ........R. .Y.......A .......... .......... .......... .........R .......... .......... W......... ..........

PK-16 ........R. .Y.......A .......... .......... .......... .........R .......... .......... W......... ..........

MM-80 ........R. .Y.......A .......... .......... .......... .........R .......... .......... W......... ..........

MM-3dg ........R. .Y.......A .......... .......... .......... .........R .......... .......... W......... ..........

TH-88 ........R. .Y.......A .......... .......... .......... .........R .......... .......... W......... ..........

MM-4 ........G. .Y.......A .......... .......... .......... .........R .......... .......... W......... ..........

TH-87 ........R. .Y.......A .......... .......... .......... .........R .......... .......... W......... ..........

Jatsi-109 ........R. .Y.......A .......... .......... .......... .........R .......... .......... W......... ..........

Norna-22 ........G. .C.......A .......... .......... .......... .........A .......... .......... T......... ..........

Heija-28 ........G. .C.......A .......... .......... .......... .........A .......... .......... T......... ..........

HK-23 ........G. .C.......A .......... .......... .......... .........A .......... .......... T......... ..........

Ville-37 ........G. .C.......A .......... .......... .......... .........A .......... .......... T......... ..........

HK-24 ........G. .C.......A .......... .......... .......... .........A .......... .......... T......... ..........

Heisa-33 ........G. .C.......A .......... .......... .......... .........A .......... .......... .......... ..........

Heisa-34 ........G. .C.......A .......... .......... .......... .........A .......... .......... .......... ..........

IS-39 .......... .C.......A .......... ....C...A. .......... .......... .......... .......... .......... ........A.

IS-40 .......... .C.......A .......... ....C...A. .......... .......... .......... .......... .......... ........A.

Z23-97 ........G. .........A ...A...... .....C..A. .A.....C.. ......A... ..T.....A. .C........ C........T ..T..C..A.

Z23-98 ........G. .........A ...A...... .....C..A. .A.....C.. ......A... ..T.....A. .C........ C........T ..T..C..A.

Mu-81 ........G. .........A ...A...... .....C..A. .A.....C.. ......A... ..T.....A. .C........ C........T ..T..C..A.

Mu-83 ........G. .........A ...A...... .....C..A. .A.....C.. ......A... ..T.....A. .C........ C........T ..T..C..A.

RYNV-BS .......... .C.......A ...A.....C ....CG..A. ....A..C.. ......T... ..T.....A. .C.....G.. C.....A... .....C..A.

RYNV .......... .C.......A ...A.....C ....CG..A. ....A..C.. ......T... ..T.....A. .C.....G.. C.....A... .....C..A.

Consensus ........a. .c.......a ...c.....g ....gt..a. .g..c..t.. ......c..g ..a.....t. .t.....a.. ......c..a ..c..t..a.

301 400

RYNV-Ca GGAAGCCCAA CAAAGCAGAC TCAGCTGGCA AGGAAGAAAT CTGCGCTTAC GCAAGCGGTA AGTTCCCAAC GGTGAAATCT ACCATTGGCG CAGAAATCTT

MM-79 .......... .......... .......... .......... ......W... .......... .......R.. R......... .......A.. ..........

MM-78 .......... .......... .......... .......... ......W... .......... .......R.. R......... .......A.. ..........

PK-16 .......... .......... .......... .......... ......W... .......... .......R.. R......... .......A.. ..........

MM-80 .......... .......... .......... .......... ......W... .......... .......R.. R......... .......A.. ..........

MM-3dg .......... .......... .......... .......... ......W... .......... .......R.. R......... .......A.. ..........

TH-88 .......... .......... .......... .......... ......W... .......... .......R.. R......... .......A.. ..........

MM-4 .......... .......... .......... .......... ......W... .......... .......R.. R......... .......A.. ..........

TH-87 .......... .......... .......... .......... ......W... .......... .......R.. R......... .......A.. ..........

Jatsi-109 .......... .......... .......... .......... ......W... .......... .......R.. R......... .......A.. ..........

Norna-22 .......... .......... .......... .......... ......A... .......... .......G.. A......... .......A.. ..........

Heija-28 .......... .......... .......... .......... ......A... .......... .......G.. A......... .......A.. ..........

HK-23 .......... .......... .......... .......... ......A... .......... .......G.. A......... .......A.. ..........

Ville-37 .......... .......... .......... .......... ......A... .......... .......G.. A......... .......A.. ..........

HK-24 .......... .......... .......... .......... ......A... .......... .......G.. A......... .......A.. ..........

Heisa-33 .......... .......... .......... .......... ......A... .......... .......G.. A......... .......A.. ..........

Heisa-34 .......... .......... .......... .......... ......A... .......... .......G.. A......... .......A.. ..........

IS-39 .......... ...G...... .....A..A. .......... ......A..T .....T..G. .A........ ...C.....A ..T....A.. ..........

IS-40 .......... ...G...... .....A..A. .......... ......A..T .....T..G. .A........ ...C.....A ..T....A.. ..........

Z23-97 .......... .......... .....A.... .A..G..... ...T..C... .....T..G. .......C.. .........A .......A.. ..........

Z23-98 .......... .......... .....A.... .A..G..... ...T..C... .....T..G. .......C.. .........A .......A.. ..........

Mu-81 .......... .......... .....A.... .A..G..... ...T..C... .....T..G. .......C.. .........A .......A.. ..........

Mu-83 .......... .......... .....A.... .A..G..... ...T..C... .....T..G. .......C.. .........A .......A.. ..........

RYNV-BS .........T G..G...... .....AA... .......... .......... ..C..T.... .A.....C.. ...A.....A ..A..A.A.. ..........

RYNV .........T G..G...... .....AA... .......... .......... ..C..T.... .A.....C.. ...A.....A ..A..A.A.. ....G.....

Consensus .........a c..a...... .....ag.c. .g..a..... ...c.....c ..a..t..t. .g........ g..g.....a ..c..t.a.. ....a.....

401 500

RYNV-Ca CGCTGTAATG GAGTCCTTAG AAAAATTTAA AATTTTCTAC ATGAACAAGG ACGAGATCAC CATCAGGACC GACTGCCACG CCATCATCAC CTTCTATGAA

MM-79 ...G...... .......... .......C.. .......... .....T.... .......... ......A... ..T....... .......... ...T..C...

MM-78 ...G...... .......... .......C.. .......... .....T.... .......... ......A... ..T....... .......... ...T..C...

PK-16 ...G...... .......... .......C.. .......... .....T.... .......... ......A... ..T....... .......... ...T..C...

MM-80 ...G...... .......... .......C.. .......... .....T.... .......... ......A... ..T....... .......... ...T..C...

MM-3dg ...G...... .......... .......C.. .......... .....T.... .......... ......A... ..T....... .......... ...T..C...

TH-88 ...G...... .......... .......C.. .......... .....T.... .......... ......A... ..T....... .......... ...T..C...

MM-4 ...G...... .......... .......C.. .......... .....T.... .......... ......A... ..T....... .......... ...T..C...

TH-87 ...G...... .......... .......C.. .......... .....T.... .......... ......A... ..T....... .......... ...T..C...

Jatsi-109 ...G...... .......... .......C.. .......... .....T.... .......... ......A... ..T....... ........T. ...T..C...

Norna-22 ...G...... .......... .......C.. .......... .....T.... .......... ......A... ..T....... .......... ...T..C...

Heija-28 ...G...... .......... .......C.. .......... .....T.... .......... ......A... ..T....... .......... ...T..C...

HK-23 ...G...... .......... .......C.. .......... .....T.... .......... ......A... ..T....... .......... ...T..C...

Ville-37 ...G...... .......... .......C.. .......... .....T.... .......... ......A... ..T....... .......... ...T..C...

HK-24 ...G...... .......... .......C.. .......... .....T.... .......... ......A... ..T....... .......... ...T..C...

Heisa-33 ...G...... .......... .......C.. .......... .....T.... .......... ......A... ..T....... .......... ...T..C...

Heisa-34 ...G...... .......... .......C.. .......... .....T.... .......... ......A... ..T....... .......... ...T..C...

IS-39 ...G..C... ..A.....G. .......C.. G..A..T... .......... .T........ .......... .......... .......A.. ...T..C..G

IS-40 ...G..C... ..A.....G. .......C.. G..A..T... .......... .T........ .......... .......... .......A.. ...T..C..G

Z23-97 ...G...... .......... .......... .......... .......... ....AG.... ....C.A..T .......... .......T.. ......C..G

Z23-98 ...G...... .......... .......... .......... .......... ....AG.... ....C.A..T .......... .......T.. ......C..G

Mu-81 ...G...... .......... .......... .......... .......... ....AG.... ....C.A..T .......... .......T.. ......C..G

Mu-83 ...G...... .......... .......... .......... .......... ....AG.... ....C.A..T .......... .......T.. ......C..G

RYNV-BS ...A..T... ........G. .......C.. G.....T... .......... .....G.... T........T ..T..T..A. .A..A..... ......C..G

RYNV ...A..T... ........G. .......C.. G.....T... .......... .....G.... .........T ..T..T..A. .A..A..... ......C..G

Consensus ...g..a... ..g.....a. .......c.. a..t..c... .....c.... .c..ga.... c...a.g..c ..t..c..c. .c..c..c.. ...c..c..g

501 559

RYNV-Ca AAGTTAAACG CCAAGAAACC TTCTCGGGTA AGGTGGTTAG CTTTTTGTGA TTATATAAC

MM-79 .......... .......... .......... .......... .......... .........

MM-78 .......... .......... .......... .......... .......... .........

PK-16 .......... .......... .......... .......... .......... .........

MM-80 .......... .......... .......... .......... .......... .........

MM-3dg .......... .......... .......... .......... .......... .........

TH-88 .......... .......... .......... .......... .......... .........

MM-4 .......... .......... .......... .......... .......... .........

TH-87 .......... .......... .......... .......... .......... .........

Jatsi-109 .......... .......... .......... .......... .......... .........

Norna-22 .......... .......... .......... .......... .......... .........

Heija-28 .......... .......... .......... .......... .......... .........

HK-23 .......... .......... .......... .......... .......... .........

Ville-37 .......... .......... .......... .......... .......... .........

HK-24 .......... .......... .......... .......... .......... .........

Heisa-33 .......... .......... .......... .......... .......... .........

Heisa-34 .......... .......... .......... .......... .......... .........

IS-39 .......... .......G.. ...A..C... .......... .......C.. C........

IS-40 .......... .......G.. ...A..C... .......... .......C.. C........

Z23-97 .......... .......... ....A..... .......... .......C.. C........

Z23-98 .......... .......... ....A..... .......... .......C.. C........

Mu-81 .......... .......... ....A..... .......... .......C.. C........

Mu-83 .......... .......... ....A..... .......... .......C.. C........

RYNV-BS ...C.G..T. .A........ ...GA..... ......C.T. .C.....C.. .........

RYNV ...C.G..T. .A........ ...GA..... ......C.T. .C.....C.. .........

Consensus ...t.a..c. .c.....a.. ...tc.g... ......t.a. .t.....c.. t........
