# Supplementary material for: Small-RNA analysis of pre-basic mother plants and conserved accessions of plant genetic resources for the presence of viruses
Source: PLoS One. 2019 Aug 7;14(8):e0220621. doi: 10.1371/journal.pone.0220621 (PMC6685626; doi:10.1371/journal.pone.0220621)
Supplement: S2 Fig — Each nucleotide sequence indicates sequence from an independent PCR clone of MM-3dg. The uppermost sequence is the sequence obtained by direct sequencing of the PCR product. All sequences are different to each other except the sequences of MM-3-2 and MM-3-3, and MM-3-1 and MM-3-7 are identical to each other. (DOCX) [file pone.0220621.s002.docx]

**S2 Figure.** **Multiple alignment of nucleotide sequences of the open reading frame 3 (ORF3) genomic region of ten PCR clones of rubus yellow net virus (RYNV) isolate MM-3dg.** Each nucleotide sequence indicates sequence from an independent PCR clone of MM-3dg. The uppermost sequence is the sequence obtained by direct sequencing of the PCR product. All sequences are different to each other except the sequences of MM-3-2 and MM-3-3, and MM-3-1 and MM-3-7 are identical to each other.

1 100

MM-3dg *CATAGCCGTC* *TACATCGACG* *ATATCCTGGT* *CTTCAGCAAG* *ACCTT*S*AAGG* *AGCACGAAAA* *GCACCTGAGC* *ATCATGCTTG* *GGATATGTCG* *AGACAACGGC*

MM-3-1 *..........* *..........* *..........* *..........* *.....G....* *..........* *..........* *..........* *..........* *..........*

MM-3-7 *..........* *..........* *..........* *..........* *.....G....* *..........* *..........* *..........* *..........* *..........*

MM-3-2 *.........* *..........* *..........* *..........* *.....G....* *..........* *..........* *..........* *..........* *..........*

MM-3-3 *..........* *..........* *..........* *..........* *.....G....* *..........* *..........* *..........* *..........* *..........*

MM-3-5 *..........* *..........* *..........* *..........* *.....*C*....* *..........* *..........* *..........* *..........* *..........*

MM-3-4 *..........* *..........* *..........* *..........* *.....G....* *..........* *..........* *..........* *..........* *..........*

MM-3-10 *..........* *..........* *..........* *..........* *.....G....* *..........* *..........* *..........* *..........* *..........*

MM-3-9 *..........* *..........* *..........* *..........* *.....G....* *..........* *..........* *..........* *..........* *..........*

MM-3-6 *..........* *..........* *..........* *..........* *.....G....* *..........* *..........* *..........* *..........* *..........*

MM-3-8 *..........* *..........* *..........* *..........* *.....G....* *..........* *..........* *..........* *..........* *..........*

Consensus *..........* *..........* *..........* *..........* *.....g....* *..........* *..........* *..........* *..........* *..........*

101 200

MM-3dg *CTGGTTTTGT* *CACCAAGCAA* *GATGAAGTTA* *GCAGCAACCG* *AGATCGACTT* *CTTGGGAGCC* *ACCATTGGTG* *ACGGAAAGAT* *TAAACTCCAG* *CCTCACATAA*

MM-3-1 *..........* *..........* *..........* *..........* *..........* *..........* *..........* *..........* *..........* *..........*

MM-3-7 *..........* *..........* *..........* *..........* *..........* *..........* *..........* *..........* *..........* *..........*

MM-3-2 *..........* *..........* *..........* *..........* *..........* *..........* *..........* *..........* *..........* *..........*

MM-3-3 *..........* *..........* *..........* *..........* *..........* *..........* *..........* *..........* *..........* *..........*

MM-3-5 *..........* *..........* *..........* *..........* *..........* *..........* *..........* *..........* *..........* *..........*

MM-3-4 *..........* *..........* *..........* *..........* *..........* *..........* *..........* *..........* *..........* *..........*

MM-3-10 *..........* *..........* *..........* *..........* *..........* *..........* *..........* *..........* *..........* *..........*

MM-3-9 *.....*A*....* *......*CT*..* A*......*A*..* *..*T*.......* *....*A*..*T*..* *..........* *..*T*..*A*....* *......*GA*..* C*.........* *..........*

MM-3-6 *..........* *..........* *..........* *..........* *..........* *..........* *..........* *..........* *..........* *..........*

MM-3-8 *..........* *..........* *..........* *..........* *..........* *..........* *..*T*..*A*....* *......*GA*..* C*.........* *..........*

Consensus *.....t....* *......gc..* *g......t..* *..a.......* *....c..c..* *..........* *..c..t....* *......ag..* *t.........* *..........*

201 300

MM-3dg *TCAAGAA*G*AT* *AGC*T*GAGGTG* *GACGATGA*A*T* *C*T*CT*R*AA*R*AC* *CCTCAA*R*GG*G *TTGAG*A*AGTT* *GGTTGGGAGT* Y*CTCAACTA*T *GC*C*AG*R*AA*C*T* *ACAT*Y*CC*G*AA*

MM-3-1 *.......*.*..* *...*.*......* *........*.*.* *.*.*..*A*..*A*..* *......*A*..*. *.....*.*....* *..........* T*........*. *..*.*..G..*.*.* *....*C*..*.*..*

MM-3-7 *.......*.*..* *...*.*......* *........*.*.* *.*.*..*A*..*A*..* *......*A*..*. *.....*.*....* *..........* T*........*. *..*.*..G..*.*.* *....*C*..*.*..*

MM-3-2 *.......*.*..* *...*.*......* *........*.*.* *.*.*..*G*..G..* *......G..*. *.....*.*....* *..........* *C........*. *..*.*..*A*..*.*.* *....T..*.*..*

MM-3-3 *.......*.*..* *...*.*......* *........*.*.* *.*.*..*G*..G..* *......G..*. *.....*.*....* *..........* *C........*. *..*.*..*A*..*.*.* *....T..*.*..*

MM-3-5 *.......*.*..* *...*.*......* *........*.*.* *.*.*..*G*..G..* *......G..*. *.....*.G*...* *..........* *C........*. *..*.*..*A*..*.*.* *....T..*.*..*

MM-3-4 *.......*.*..* *...*.*......* *........*.*.* *.*.*..*G*..G..* *......G..*. *.....*.*....* *..........* *C........*. *..*.*..*A*..*.*.* *....*C*..*.*..*

MM-3-10 *.......*.*..* *...*.*......* *........*.*.* *.*.*..*A*..*A*..* *......*A*..*. *.....*.*....* *..........* T*........*. *..*.*..G..*.*.* *....*C*..*.*..*

MM-3-9 *.......A..* *...A......* *........G.* *.A..C..G..* *......G..A* *.....G....* *..........* *C........C* *..G..G..T.* *....T..A..*

MM-3-6 *.......A..* *...A......* *........G.* *.A..C..G..* *......G..A* *.....G....* *..........* *C........C* *..G..G..T.* *....T..A..*

MM-3-8 *.......A..* *...A......* *........G.* *.A..C..G..* *......G..A* *.....G....* *..........* *C........C* *..G..G..T.* *....T..A..*

Consensus *.......a..* *...a......* *........g.* *.a..c..g..* *......g..a* *.....g....* *..........* *c........c* *..g..g..t.* *....t..a..*

301 400

MM-3dg *GTGCGGAAC*A *CT*C*CT*R*GGCC* *C*R*CTATACAG* *CAAGAC*C*AG*Y *GAGCA*T*GGAG* *ACAG*A*AG*R*TG* *GCA*T*GC*W*TCG* *GATTGGGCCT* *TAGTAAAGAA* *GATCAA*R*AGC*

MM-3-1 *.........*. *..*.*..*A*....* *.*G*........* *......*.*..*C *.....*.*....* *....*.*..A..* *...*.*..A...* *..........* *..........* *......A...*

MM-3-7 *.........*. *..*.*..*A*....* *.*G*........* *......*.*..*C *.....*.*....* *....*.*..A..* *...*.*..A...* *..........* *..........* *......A...*

MM-3-2 *.........*. *..*.*..*G*....* *.*G*........* *......*.*..*C *.....*.*....* *....*.*..A..* *...*.*..A...* *..........* *..........* *......A...*

MM-3-3 *.........*. *..*.*..*G*....* *.*G*........* *......*.*..*C *.....*.*....* *....*.*..A..* *...*.*..A...* *..........* *..........* *......A...*

MM-3-5 *.........*. *..*.*..*G*....* *.*G*........* *......*.*..*C *.....*.*....* *....*.*..A..* *...*.*..A...* *..........* *..........* *......A...*

MM-3-4 *.........*. *..*.*..*A*....* *.*G*........* *......*.*..T* *.....*.*....* *....*.*..*G*..* *...*.*..*T*...* *..........* *..........* *......*G*...*

MM-3-10 *.........*. *..*.*..*A*....* *.A........* *......*.*..T* *.....*.*....* *....*.*..*G*..* *...*.*..*T*...* *..........* *..........* *......*G*...*

MM-3-9 *.........T* *..T..C....* *.A........* *......T..T* *.....C....* *....G..A..* *...C..A...* *..........* *..........* *......A...*

MM-3-6 *.........T* *..T..C....* *.A........* *......T..T* *.....C....* *....G..A..* *...C..A..*A *..........* *.*G*..*C*.....* *......A...*

MM-3-8 *.........T* *..T..C....* *.A........* *......T..T* *.....C....* *....G..A..* *...C..A..*A *..........* *.*G*..*C*.....* *......A...*

Consensus *.........t* *..t..c....* *.a........* *......t..t* *.....c....* *....g..a..* *...c..a..g* *..........* *.a..a.....* *......a...*

401 500

MM-3dg *CTGGTCCA*R*A* *A*Y*CTCCCAGA* *CCTCAAACTG* *CCCAGTGAGG* *AGGCCTATAT* *GATCATCGA*R *ACAGATGGTT* *GTATGGAAGG* W*TGGGGCGGA* *GTCTGTAAGT*

MM-3-1 *........G.* *.*C*........* *..........* *..........* *..........* *.........*A *..........* *..........* T*.........* *..........*

MM-3-7 *........G.* *.*C*........* *..........* *..........* *..........* *.........*A *..........* *..........* T*.........* *..........*

MM-3-2 *........G.* *.*C*........* *..........* *..........* *..........* *.........*A *..........* *..........* T*.........* *..........*

MM-3-3 *........G.* *.*C*........* *..........* *..........* *..........* *.........*A *..........* *..........* T*.........* *..........*

MM-3-5 *........G.* *.*C*........* *..........* *..........* *..........* *.........*A *..........* *..........* T*.........* *..........*

MM-3-4 *........*A*.* *.*T*........* *..........* *..........* *..........* *.........*G *..........* *..........* A*.........* *..........*

MM-3-10 *........*A*.* *.*T*........* *..........* *..........* *..........* *.........*G *..........* *..........* A*.........* *..........*

MM-3-9 *........G.* *.*C*........* *..........* *..........* *..........* *.........*A *..........* *..........* T*.........* *..........*

MM-3-6 *........G.* *.*T*........* *...*A*......* *.....*C*..*A*.* *.*A*.....*C*..* *......*A*..*G *..*T*.....*A*.* *.*C*........* C*........*T *..*T*..*C*..*A*.*

MM-3-8 *........G.* *.*T*........* *...*A*......* *.....*C*..*A*.* *.*A*.....*C*..* *......*A*..*G *..*T*.....*A*.* *.*C*........* C*........*T *..*T*..*C*..*A*.*

Consensus *........g.* *.*.*........* *...c......* *.....t..g.* *.g.....t..* *......c..*. *..a.....t.* *.t........* .*........a* *..c..t..g.*

501 600

MM-3dg *GGAAGCCCAA* *CAAAGCAGAC* *TCAGCTGGCA* *AGGAAGAAAT* *CTGCGC*W*TAC* *GCAAGCGGTA* *AGTTCCC*R*AC* R*GTGAAATCT* *ACCATTGACG* *CAGAAATCTT*

MM-3-1 *..........* *..........* *..........* *..........* *......*A*...* *..........* *.......G..* *A.........* *..........* *..........*

MM-3-7 *..........* *..........* *..........* *..........* *......*A*...* *..........* *.......G..* *A.........* *..........* *..........*

MM-3-2 *..........* *..........* *..........* *..........* *......*A*...* *..........* *.......G..* *A.........* *..........* *..........*

MM-3-3 *..........* *..........* *..........* *..........* *......*A*...* *..........* *.......G..* *A.........* *..........* *..........*

MM-3-5 *..........* *..........* *..........* *..........* *......*A*...* *..........* *.......G..* *A.........* *..........* *..........*

MM-3-4 *..........* *..........* *..........* *..........* *......*T*...* *..........* *.......*A*..* G*.........* *..........* *..........*

MM-3-10 *..........* *..........* *..........* *..........* *......*T*...* *..........* *.......*A*..* G*.........* *..........* *..........*

MM-3-9 *..........* *..........* *..........* *..........* *......*A*...* *..........* *.......G..* *A.........* *..........* *..........*

MM-3-6 *..........* *..........* *.....*A*....* *.*A*..*G*.....* *...*T*..*C*...* *..........* *.......G..* *A.........* *..........* *..........*

MM-3-8 *..........* *..........* *.....*A*....* *.*A*..*G*.....* *...*T*..*C*...* *.....*T*..*G*.* *.......*C*..* G*........*A *..........* *..........*

Consensus *..........* *..........* *.....t....* *.g..a.....* *...c..*.*...* *.....c..t.* *.......g..* *a........t* *..........* *..........*

601 700

MM-3dg *CGCGGTAATG* *GAGTCCTTAG* *AAAAATTCAA* *AATTTTCTAC* *ATGAATAAGG* *ACGAGATCAC* *CATCAGAACC* *GATTGCCACG* *CCATCATCAC* *CTTTTACGAA*

MM-3-1 *..........* *..........* *..........* *..........* *..........* *..........* *..........* *..........* *..........* *..........*

MM-3-7 *..........* *..........* *..........* *..........* *..........* *..........* *..........* *..........* *..........* *..........*

MM-3-2 *..........* *..........* *..........* *..........* *..........* *..........* *..........* *..........* *..........* *..........*

MM-3-3 *..........* *..........* *..........* *..........* *..........* *..........* *..........* *..........* *..........* *..........*

MM-3-5 *..........* *..........* *..........* *..........* *..........* *..........* *..........* *..........* *..........* *..........*

MM-3-4 *..........* *..........* *..........* *..........* *..........* *..........* *..........* *..........* *..........* *..........*

MM-3-10 *..........* *..........* *..........* *..........* *..........* *..........* *..........* *..........* *..........* *..........*

MM-3-9 *..........* *..........* *..........* *..........* *..........* *..........* *..........* *..........* *..........* *..........*

MM-3-6 *..........* *..........* *.......*T*..* *..........* *.....*C*....* *..........* *......*G*...* *..*C*.......* *..........* *...*C*..*T*...*

MM-3-8 *..........* *..........* *.......*T*..* *..........* *.....*C*....* *....*AG*....* *....*C*....*T *..*C*.......* *.......*T*..* *...*C*.....*G

Consensus *..........* *..........* *.......c..* *..........* *.....t....* *....ga....* *....a.a..c* *..t.......* *.......c..* *...t..c..a*

701 779

MM-3dg *AAGTTAAACG* *CCAAGAAACC* *TTCTCGGGTA* *AGGTGGTTAG* *CTTTTTGTGA* *TTATATAACA* *AACTCAGGGG* *TGAAGATGA*

MM-3-1 *..........* *..........* *..........* *..........* *..........* *..........* *..........* *.........*

MM-3-7 *..........* *..........* *..........* *..........* *..........* *..........* *..........* *.........*

MM-3-2 *..........* *..........* *..........* *..........* *..........* *..........* *..........* *.........*

MM-3-3 *..........* *..........* *..........* *..........* *..........* *..........* *..........* *.........*

MM-3-5 *..........* *..........* *..........* *..........* *..........* *..........* *..........* *.........*

MM-3-4 *..........* *..........* *.....*A*....* *..........* *..........* *..........* *..........* *.........*

MM-3-10 *..........* *..........* *..........* *..........* *..........* *..........* *..........* *.........*

MM-3-9 *..........* *..........* *..........* *..........* *..........* *..........* *..........* *.........*

MM-3-6 *..........* *..........* *..........* *..........* *..........* *..........* *..........* *.........*

MM-3-8 *..........* *..........* *....*A*.....* *..........* *.......*C*..* C*........*G *..........* *.........*

Consensus *..........* *..........* *....c.....* *..........* *.......t..* *t........a* *..........* *.........*
