# Supplementary material for: Small-RNA analysis of pre-basic mother plants and conserved accessions of plant genetic resources for the presence of viruses
Source: PLoS One. 2019 Aug 7;14(8):e0220621. doi: 10.1371/journal.pone.0220621 (PMC6685626; doi:10.1371/journal.pone.0220621)
Supplement: S3 Fig — Each amino acid sequence indicates sequence from an independent PCR clone of MM-3dg. The uppermost sequence is obtained by direct sequencing of the PCR product. X denotes the position containing degenerate nucleotides. The amino acid sequences of the clones 1, 2, 3, 6, 7 and 10 of MM-3dg are identical to each other, whereas the others differ from each other. There were totally five different sequences between the ten sequenced clones. (DOCX) [file pone.0220621.s003.docx]

1 100

MM-3dg *IAVYIDDILV* *FSKT*X*KEHEK* *HLSIMLGICR* *DNGLVLSPSK* *MKLAATEIDF* *LGATIGDGKI* *KLQPHIIKKI* *AEVDDES*XX*T* *L*X*GLRSWLG*X *LNYA*X*NY*X*PK*

MM-3-1 *..........* *....*L*.....* *..........* *..........* *..........* *..........* *..........* *.......LK.* *.K.......V* *....R..I..*

MM-3-2 *..........* *....*L*.....* *..........* *..........* *..........* *..........* *..........* *.......LK.* *.K.......V* *....R..I..*

MM-3-10 *..........* *....*L*.....* *..........* *..........* *..........* *..........* *..........* *.......LK.* *.K.......V* *....R..I..*

MM-3-3 *..........* *....*L*.....* *..........* *..........* *..........* *..........* *..........* *.......LK.* *.K.......V* *....R..I..*

MM-3-6 *..........* *....*L*.....* *..........* *..........* *..........* *..........* *..........* *.......LK.* *.K.......V* *....R..I..*

MM-3-7 *..........* *....*L*.....* *..........* *..........* *..........* *..........* *..........* *.......LK.* *.K.......V* *....R..I..*

MM-3-8 *..........* *....*L*.....* *..........* *..........* *..........* *........*R*.* *..........* *.......LK.* *.K.......V* *....R..I..*

MM-3-4 *..........* *....*L*.....* *..........* *..........* *..........* *..........* *..........* *.......LK.* *.K.......V* *....R..I..*

MM-3-9 *..........* *....*L*.....* *..........* *........*T*.* *..*I*.......* *........*R*.* *..........* *.......LK.* *.K.......V* *....R..I..*

MM-3-5 *..........* *....*F*.....* *..........* *..........* *..........* *..........* *..........* *.......LK.* *.K...*G*...V* *....R..I..*

Consensus *..........* *....*.*.....* *..........* *........s.* *..l.......* *........k.* *..........* *.......lk.* *.k...s...v* *....r..i..*

101 200

MM-3dg *CGTL*X*G*X*LYS* *KT*X*EHGDR*X*W* *H*X*SDWALVKK* *I*X*SLV*XX*LPD* *LKLPSEEAYM* *II*X*TDGCME*X *WGGVCKWKPN* *KADSAGKEEI* *C*X*YASGKF*XX *VKSTIDAEIF*

MM-3-1 *....L.P...* *..S.....R.* *.A........* *.K...QN...* *..........* *..E......G* *..........* *..........* *.A......PT* *..........*

MM-3-2 *....L.P...* *..S.....R.* *.A........* *.K...QN...* *..........* *..E......G* *..........* *..........* *.A......PT* *..........*

MM-3-10 *....L.P...* *..S.....R.* *.A........* *.K...QN...* *..........* *..E......G* *..........* *..........* *.A......PT* *..........*

MM-3-3 *....L.P...* *..S.....R.* *.A........* *.K...QN...* *..........* *..E......G* *..........* *..........* *.A......PT* *..........*

MM-3-6 *....L.P...* *..S.....R.* *.A........* *.K...QN...* *..........* *..E......G* *..........* *..........* *.A......PT* *..........*

MM-3-7 *....L.P...* *..S.....R.* *.A........* *.K...QN...* *..........* *..E......G* *..........* *..........* *.A......PT* *..........*

MM-3-8 *....L.P...* *..S.....R.* *.A........* *.K...QN...* *..........* *..E......G* *..........* *..........* *.A......PT* *..........*

MM-3-4 *....L.P...* *..S.....R.* *.A........* *.K...QN...* *..........* *..E......G* *..........* *..........* *.A......PT* *..........*

MM-3-9 *....L.P...* *..S.....R.* *.A........* *.K...QN...* *..........* *..E......G* *..........* *..........* *.A......PT* *..........*

MM-3-5 *....L.P...* *..S.....R.* *.A........* *.K...QN...* *..........* *..E......G* *..........* *..........* *.A......PT* *..........*

Consensus *....l.p...* *..s.....r.* *.a........* *.k...qn...* *..........* *..e......g* *..........* *..........* *.a......pt* *..........*

201 259

MM-3dg *AVMESLEKFK* *IFYMNKDEIT* *IRTDCHAIIT* *FYEKLNAKKP* *SRVRWLAFCD* *YITNSGVKM*

MM-3-1 *..........* *..........* *..........* *..........* *..........* *.........*

MM-3-2 *..........* *..........* *..........* *..........* *..........* *.........*

MM-3-10 *..........* *..........* *..........* *..........* *..........* *.........*

MM-3-3 *..........* *..........* *..........* *..........* *..........* *.........*

MM-3-6 *..........* *..........* *..........* *..........* *..........* *.........*

MM-3-7 *..........* *..........* *..........* *..........* *..........* *.........*

MM-3-8 *..........* *........V.* *..........* *..........* *..........* *.........*

MM-3-4 *..........* *..........* *..........* *..........* *.*Q*........* *.........*

MM-3-9 *..........* *..........* *..........* *..........* *..........* *.........*

MM-3-5 *..........* *..........* *..........* *..........* *..........* *.........*

Consensus *..........* *..........* *..........* *..........* *..........* *.........*

**S3 Figure. Multiple alignment of amino acid sequences of the open reading frame 3 (ORF3) of ten PCR clones of rubus yellow net virus (RYNV) isolate MM-3dg.** Each amino acid sequence indicates sequence from an independent PCR clone of MM-3dg. The uppermost sequence is obtained by direct sequencing of the PCR product. X denotes the position containing degenerate nucleotides. The amino acid sequences of the clones 1, 2, 3, 6, 7 and 10 of MM-3dg are identical to each other, whereas the others differ from each other. There were totally five different sequences between the ten sequenced clones.
