# Supplementary material for: Small-RNA analysis of pre-basic mother plants and conserved accessions of plant genetic resources for the presence of viruses
Source: PLoS One. 2019 Aug 7;14(8):e0220621. doi: 10.1371/journal.pone.0220621 (PMC6685626; doi:10.1371/journal.pone.0220621)
Supplement: S4 Fig — Only the nucleotides that differ from those of RYNV-Ca are shown. Identical nucleotides are indicated by dots. The aligned region corresponds to the nucleotides 6081–7920 and nucleotides 1–496 of the complete nucleotide sequence of RYNV-Ca. The open reading frames encoded by the sequences are indicated. 5’-terminal part of ORF1 in green, 3’-terminal part of ORF3 in bold and underlined, ORF4 in blue, ORF5 in red and ORF7 encoded in antisense strand in cursive and underlined. (DOCX) [file pone.0220621.s004.docx]

**1 100**

**…ORF3**

**RYNV-Ca TCATAGCCGT CTACATCGAC GATATCCTGG TCTTCAGCAA GACCTTGAAG GAGCACGAAA AGCACCTGAG CATCATGCTT GGGATATGTC GAGACAACGG**

**MM-3 .......... .......... .......... .......... .......... .......... .......... .......... .......... ..........**

**Consensus .......... .......... .......... .......... .......... .......... .......... .......... .......... ..........**

**101 200**

**RYNV-Ca CCTGGTTTTG TCACCAAGCA AGATGAGGTT AGCAGCAACC GAGATCGACT TCTTGGGAGC CAGCATTGGT GACGGAAAGA TTAAACTCCA GCCTCACATA**

**MM-3 .......... .......... ......A... .......... .......... .......... ..C....... .......... .......... ..........**

**Consensus .......... .......... ......a... .......... .......... .......... ..c....... .......... .......... ..........**

**201 300**

**RYNV-Ca ATCAAGAAGA TAGCTGAGGT GGACGATGAA TCTCTGAAGA CCCTCAAGGG GCTGAGAAGT TGGTTGGGAG TTCTCAACTA TGCCAGGAAC TACATCCCGA**

**MM-3 .......... .......... .......... ........A. .......A.. .T........ .......... .......... .......... ..........**

**Consensus .......... .......... .......... ........a. .......a.. .c........ .......... .......... .......... ..........**

**301 400**

**RYNV-Ca AGTGCGGAAC ACTCCTAGGC CCACTATACA GCAAGACCAG TGAGCATGGA GACAGAAGGT GGCATGCTTC GGATTGGGCC TTAGTAAAGA AGATCAAGAG**

**MM-3 .......... .......... ..G....... .......... C......... ........A. .......A.. .......... .......... .......A..**

**Consensus .......... .......... ..a....... .......... c......... ........a. .......a.. .......... .......... .......a..**

**401 500**

**RYNV-Ca CCTGGTCCAA AATCTCCCAG GCCTCAAACT GCCCAGTGAG GAGGCCTATA TGATCATCGA GACAGATGGT TGTATGGAAG GATGGGGCGG AGTCTGTAAG**

**MM-3 .........G ..C....... A......... .......... .......... .......... A......... .......... .......... ..........**

**Consensus .........a ..c....... a......... .......... .......... .......... a......... .......... .......... ..........**

**501 600**

**RYNV-Ca TGGAAGCCCA ACAAAGCAGA CTCAGCTGGC AAGGAAGAAA TCTGCGCTTA CGCAAGCGGT AAGTTCCCAA CGGTGAAATC TACCATTGGC GCAGAAATCT**

**MM-3 .......... .......... .......... .......... .......A.. .......... ........G. .A........ ........A. ..........**

**Consensus .......... .......... .......... .......... .......a.. .......... ........a. .a........ ........a. ..........**

**601 700**

**RYNV-Ca TCGCTGTAAT GGAGTCCTTA GAAAAATTTA AAATTTTCTA CATGAACAAG GACGAGATCA CCATCAGGAC CGACTGCCAC GCCATCATCA CCTTCTATGA**

**MM-3 ....G..... .......... ........C. .......... ......T... .......... .......A.. ...T...... .......... ....T..C..**

**Consensus ....g..... .......... ........c. .......... ......c... .......... .......a.. ...c...... .......... ....c..c..**

**701 800**

**RYNV-Ca AAAGTTAAAC GCCAAGAAAC CTTCTCGGGT AAGGTGGTTA GCTTTTTGTG ATTATATAAC AAACTCAGGG GTGAAGATGA AGTTCGAACA CATCAAAGGC**

**MM-3 .......... .......... .......... .......... .......... .......... .......... .......... .......... ..........**

**Consensus .......... .......... .......... .......... .......... .......... .......... .......... .......... ..........**

ORF4

**801 900**

**RYNV-Ca AAAGATAATC AGCTCGCTGA CAATCTTAGT CGCTTTACCC AACTCATCAC TGTAGTAAGA TGGCTTCCCA AGGAACTAGC GGAGCTCACG GCCGAACTGG**

**MM-3 .......... .......... .......... ...C...... .......... .......... .......... .......... .......... ..........**

**Consensus .......... .......... .......... ...c...... .......... .......... .......... .......... .......... ..........**

**901 1000**

**RYNV-Ca TCAAAGGAAG GGACGAAGCC CTGGTGAACA AGGAGGTACA GAGGAACATC TCATGTTTTC TCGAGACTGC CCTCCTCCAA GCGGAGAAAT CCGTGACTAC**

**MM-3 .......... .......... .......... .......... .......... .......... .......... .......... .......... ..........**

**Consensus .......... .......... .......... .......... .......... .......... .......... .......... .......... ..........**

**1001 1100**

**RYNV-Ca TCGCCCATCA GAGCCGCACC ATGTACTATG GCGGAGATGG ACGAATCCCG AAGAGTGGCC ATGCAGCGAA GAATCAAGGT CTTCGACGAT CTTGCACAAA**

**MM-3 .......... .......... .......... .......... .......... .......... .......... .......... .......... ..........**

**Consensus .......... .......... .......... .......... .......... .......... .......... .......... .......... ..........**

**1101 1200**

**RYNV-Ca ACATCAGCGA CGCCGTATAC ATCACAGGCA TCGACCTCGC CGCCGCCAAA GCACGGGCAA CCAGGGATAA CTGGTACAAT GACGTCACCC CGGCATTGGA**

**MM-3 .......... .......... .......... .......... .......... .......... .......... .......... .......... ..........**

**Consensus .......... .......... .......... .......... .......... .......... .......... .......... .......... ..........**

1201 1300

**RYNV-Ca AGAACGAGCA GCTGCAGCAT GGAGACTCAT GGCAGCCTAC TCAGACTTCG CCACGTGGAA GGACGTGA**AC GTCTAGTGAA GTGACGCAAG GAATGACTTC

**MM-3 .......... .......... .......... .......... .......... .......... .......**... .......... .......... ..........

**Consensus .......... .......... .......... .......... .......... .......... ........**.. .......... .......... ..........

1301 1400

RYNV-Ca ACAATTGCCA ATGTCGTCAC TGCTTACGAC TTGGAACTTA TCCTTTAGTG TCGGCAGCAT CTCTTAGCTG TCATAAGTGT GTAAGTGCGC CAGTAGTGCG

MM-3 .......... .......... .......... .......... .......... .......... .......... .......... .......... ..........

Consensus .......... .......... .......... .......... .......... .......... .......... .......... .......... ..........

*ORF7 (minus strand)*

1401 1500

RYNV-Ca CTGTGTCAAG ATAAGGAATC TTATCTCCTT ATCTTCTTTC CCTTTGT*TTA AAGGTAAAGC TGTAAAGCAG GACTAATTAG CTGCAGGTCA TCAGGTTTGC*

MM-3 .......... .......... .......... .......... .......*... .......... .......... .......... .......... ..........*

Consensus .......... .......... .......... .......... .......*... .......... .......... .......... .......... ..........*

ORF5

*1501 1600*

*RYNV-Ca GGTTGTGGAA CTCCTGCAGC TGACTGGTGA GCTCTTCGAC TTTTCTAGTG AGGAAAGCGT TGTGCTCATA GAGATGTCGT ATAAGCTCAT CTTTGTTGTG*

*MM-3 .......... .......... .......... .......... .......... .......... .......... .......... .......... ..........*

*Consensus .......... .......... .......... .......... .......... .......... .......... .......... .......... ..........*

*1601 1700*

*RYNV-Ca GTATTGCCAC TTAGCTGCGT CCTTGGTGTC CTTTGCGGCT ATAAGCTTGA TCCCATGATC CATGTATGCG CAAAGGGGAC AGAGGTTGAG GTTGCAGGTG*

*MM-3 .......... .......... .......... .......... .......... .......... .......... .......... .......... ..........*

*Consensus .......... .......... .......... .......... .......... .......... .......... .......... .......... ..........*

*1701 1800*

*RYNV-Ca GTGCAGGTGA CTCTTCGCCC ATGAGGCGTT TCGTCGCTGC ATATGCTGCA TATCCTCCCT TCGATCGGCA CTTCTTGTGT ATCACTCCAG GCGTGAGTGC*

*MM-3 .......... .......... .......... .......... .......... .......... .......... .......... .......... ..........*

*Consensus .......... .......... .......... .......... .......... .......... .......... .......... .......... ..........*

RYNV-Ca nucleotide 1->

*1801*  1900

*RYNV-Ca ATTCTTGCTG TGCCTTTGGT ATCTCCTTCC TTCTTCTCCA GGAAGGTTTT TCTGGTATCA GAGCTTTAGC TCTCACCAT*G GCAGCTTAAA CACTTCCCTT

*MM-3 .......... .........G .......... .......... .......... .......... .......... .........*. .T........ .....T....

*Consensus .......... .........g .......... .......... ..........* .......... .......... .......... .c........ .....c....

1901 2000

RYNV-Ca CTTGTCGAGA AACCCAAGTT TCAGACCAGA ACCTTGAGTT TGCTCTCTTT TTCGGAGGGA AGAGGAGTGA GTGTCTGTGT CAAAACCTTG AAAGATCAAA

MM-3 .......... .......... .......... .......... .......... .......... .......... .......... .......... ..........

Consensus .......... .......... .......... .......... .......... .......... .......... .......... .......... ..........

2001 2100

RYNV-Ca CCCCCATGAA AACTTTCCTC ACGGTACCAT GAGTTTTCTA TCCTTCACTA GTTTGAACCT ACTGCTCAAA CTGCAGGCTT AGGCGTCGAA GCGAAGTACC

MM-3 .......... .......... .......... A......... .......... .......... .......... .......... .......... ..........

Consensus .......... .......... .......... a......... .......... .......... .......... .......... .......... ..........

2101 2200

RYNV-Ca CTTGTAGCCG TTAGCAGGAG GCGTTAGGCG TTGATTGGGG AAAACTGACG TAAAGAAGCA GCAGCAACTA GGCAAGAAAC CTGACGGGTA GATCACCGGC

MM-3 .......... .......... .......... .......... .......... .......... .......... .......... .......... ..........

Consensus .......... .......... .......... .......... .......... .......... .......... .......... .......... ..........

ORF1>

2201 2300

RYNV-Ca CGGAAAGCCA GTAAGCGGCT AGATCTGGGC AGTTTTGATG CAACCTCACG AAATCTCAGC CTTCGAAGAA GAAAGCAGCT CTTGGGAAAG GTCTGAACGG

MM-3 .......... .......... .......... .......... .......... .......... .......... .......... .......... ..........

Consensus .......... .......... .......... .......... .......... .......... .......... .......... .......... ..........

2301 2348

RYNV-Ca GCGTATCGAC AAGACTTTTT ATTCAGAAAT CTCAGAACGT ATCCACGT

MM-3 .......... .......... .......... .......... ........

Consensus .......... .......... .......... .......... ........

Consensus .......... .......... .......... .......... ........

**S4 Figure. Nucleotide alignment and genomic organization of Finnish rubus yellow net virus (RYNV) isolate MM-3 relative to the previously identified sequence of RYNV-Ca (KF241951).** Only the nucleotides that differ from those of RYNV-Ca are shown. Identical nucleotides are indicated by dots. The aligned region corresponds to the nucleotides 6081-7920 and nucleotides 1-496 of the complete nucleotide sequence of RYNV-Ca. The open reading frames encoded by the sequences are indicated. 5’-terminal part of ORF1 in green, 3’-terminal part of ORF3 in bold and underlined, ORF4 in blue, ORF5 in red and ORF7 encoded in antisense strand in cursive and underlined.
