# Supplementary material for: Small-RNA analysis of pre-basic mother plants and conserved accessions of plant genetic resources for the presence of viruses
Source: PLoS One. 2019 Aug 7;14(8):e0220621. doi: 10.1371/journal.pone.0220621 (PMC6685626; doi:10.1371/journal.pone.0220621)
Supplement: S5 Fig — A, Multiple alignment of nucleotide sequences. Only nucleotides that differ from those of RYNV-Ca are shown. Identical nucleotides are indicated by dots. The aligned region corresponds to the nucleotides 6282–6840 of the complete nucleotides sequence of RYNV-Ca. B, Multiple alignment of amino acid sequences. Only amino acids that differ from those of RYNV-Ca are shown. Identical amino acids are indicated by dots. (DOCX) [file pone.0220621.s005.docx]

**A**

1 100

RYNV-Ca TCAAGAAGAT AGCTGAGGTG GACGATGAAT CTCTGAAGAC CCTCAAGGGG CTGAGAAGTT GGTTGGGAGT TCTCAACTAT GCCAGGAACT ACATCCCGAA

RYNV-BS .A........ ...C...... ..T..C.... .C.....A.. ......A... T.AC....C. .......... G........C ..GC.C.... .......A..

Krus-11 .......... C.....A..A ...A.C..G. .C..C..A.. ...G..A..A ..C..G.... ..C.T..... .........C ..A.....T. .T..T..T..

Krus-12 .......... C.....A..A ...A.C..G. .C..C..A.. ...G..A..A ..C..G.... ..C.T..... .........C ..A.....T. .T..T..T..

HY-20 .......... C.....A..A ...A.C..G. .C..C..A.. ...G..A..A ..C..G.... ..C.T..... .........C ..A.....T. .T..T..T..

Consensus .c........ a..t..g..g ..cg.c..a. .c..g..a.. ...c..a..g c..a.a..t. ..t.g..... t........c ...a.g..c. .c..c.....

101 200

RYNV-Ca GTGCGGAACA CTCCTAGGCC CACTATACAG CAAGACCAGT GAGCATGGAG ACAGAAGGTG GCATGCTTCG GATTGGGCCT TAGTAAAGAA GATCAAGAGC

RYNV-BS ...T..C... ..GT...... .......... .........C ........T. .TC.T..... ...C..G..T .......... ....C..A.G A..T...G..

Krus-11 A........C T.A..C.... ....C..... ......AGC. ..A..C.... .TC.C..A.. ....C.C... ..C....AA. ....CG.... ...T....AG

Krus-12 A........C T.A..C.... ....C..... ......AGC. ..A..C.... .TC.C..A.. ....C.C... ..C....AA. ....CG.... ...T....AG

HY-20 A........C T.A..C.... ....C..... ......AGC. ..A..C.... .TC.C..A.. ....C.C... ..C....AA. ....CG.... ...T....AG

Consensus g..c..a..a c..c.a.... ....a..... ......cagt ..g..t..a. .tc....g.. ...tg....g ..t....cc. ....ca.g.a g..t...agc

201 300

RYNV-Ca CTGGTCCAAA ATCTCCCAGG CCTCAAACTG CCCAGTGAGG AGGCCTATAT GATCATCGAG ACAGATGGTT GTATGGAAGG ATGGGGCGGA GTCTGTAAGT

RYNV-BS .......... .C.......A ...A.....C ....CG..A. ....A..C.. ......T... ..T.....A. .C.....G.. C.....A... .....C..A.

Krus-11 AAA.....GG .........A ...T..G..C ..TGCG..AA .T..T..C.. C.....A..A .....C..A. .......C.. T.....A..C ........A.

Krus-12 AAA.....GG .........A ...T..G..C ..TGCG..AA .T..T..C.. C.....A..A .....C..A. .......C.. T.....A..C ........A.

HY-20 AAA.....GG .........A ...T..G..C ..TGCG..AA .T..T..C.. C.....A..A .....C..A. .......C.. T.....A..C ........A.

Consensus ctg.....aa .t.......a ......a..c ..cacg..ag .g.....c.. g........g ..a..t..a. .t........ ......a..a .....t..a.

301 400

RYNV-Ca GGAAGCCCAA CAAAGCAGAC TCAGCTGGCA AGGAAGAAAT CTGCGCTTAC GCAAGCGGTA AGTTCCCAAC GGTGAAATCT ACCATTGGCG CAGAAATCTT

RYNV-BS .........T G..G...... .....AA... .......... .......... ..C..T.... .A.....C.. ...A.....A ..A..A.A.. ..........

Krus-11 .....AAG.G ...G..T... ATCAGA..AT C......G.. ......C..T .....T..G. ...A.T.TGT CCCA.....C ..T....ATA GC..G..G..

Krus-12 .....AAG.G ...G..T... ATCAGA..AT C......G.. ......C..T .....T..G. ...A.T.TGT CCCA.....C ..T....ATA GC..G..G..

HY-20 .....AAG.G ...G..T... ATCAGA..AT C......G.. ......C..T .....T..G. ...A.T.TGT CCCA.....C ..T....ATA GC..G..G..

Consensus .....ccc.. c..g..a... tcagcag.ca a......a.. ......t..c ..a..t..t. .g.t.c..ac ggta...... .....t.acg ca..a..c..

401 500

RYNV-Ca CGCTGTAATG GAGTCCTTAG AAAAATTTAA AATTTTCTAC ATGAACAAGG ACGAGATCAC CATCAGGACC GACTGCCACG CCATCATCAC CTTCTATGAA

RYNV-BS ...A..T... ........G. .......C.. G.....T... .......... .....G.... T........T ..T..T..A. .A..A..... ......C..G

Krus-11 T..C..C... ..AAGT..G. C...G...CG ...C.AT... ...G..G.A. TG..A..... ....C.A..A .....TA.T. .G.....T.. ......C..G

Krus-12 T..C..C... ..AAGT..G. C...G...CG ...C.AT... ...G..G.A. TG..A..... ....C.A..A .....TA.T. .G.....T.. ......C..G

HY-20 T..C..C... ..AAGT..G. C...G...CG ...C.AT... ...G..G.A. TG..A..... ....C.A..A .....TA.T. .G.....T.. ......C..G

Consensus c......... ..gtcc..g. a...a..taa a..t.tt... ...a..a.g. ac..ga.... c...a.g... ..c..tc... ....c..c.. ......c..g

501 559

RYNV-Ca AAGTTAAACG CCAAGAAACC TTCTCGGGTA AGGTGGTTAG CTTTTTGTGA TTATATAAC

RYNV-BS ...C.G..T. .A........ ...GA..... ......C.T. .C.....C.. .........

Krus-11 ...C.G..T. .A..A.G... A..AA..... ..A...C.GA G...C..... .........

Krus-12 ...C.G..T. .A..A.G... A..AA..... ..A...C.GA G...C..... .........

HY-20 ...C.G..T. .A..A.G... A..AA..... ..A...C.GA G...C..... .........

Consensus ...c.g..t. .a..g.a... t...a..... ..g...c..g ct..t..t.. .........

**B**

1 100

RYNV-Ca KKIAEVDDES LKTLKGLRSW LGVLNYARNY IPKCGTLLGP LYSKTSEHGD RRWHASDWAL VKKIKSLVQN LPGLKLPSEE AYMIIETDGC MEGWGGVCKW

RYNV-BS .......... .......... .......... .......... .......... .......... ..R..G.... ..D....T.. .......... ..........

Krus-11 .......N.. .......... .......... .......... .....A.... ....P...E. .E...KK..D ..D....A.N ..I....... .D........

Krus-12 .......N.. .......... .......... .......... .....A.... ....P...E. .E...KK..D ..D....A.N ..I....... .D........

HY-20 .......N.. .......... .......... .......... .....A.... ....P...E. .E...KK..D ..D....A.N ..I....... .D........

Consensus .......#.. .......... .......... .......... .....a.... ....p...e. .ek..kk..# ..d....a.# ..i....... .#........

101 185

RYNV-Ca KPNKADSAGK EEICAYASGK FPTVKSTIGA EIFAVMESLE KFKIFYMNKD EITIRTDCHA IITFYEKLNA KKPSRVRWLA FCDYI

RYNV-BS ..M.....S. .......... ........D. .......... .......... .V......Q. .......... .......... .....

Krus-11 .KS...IR.S .......... YSVP....DS .M.......A ..R.Y..DEV ........N. .......... .R.......S .....

Krus-12 .KS...IR.S .......... YSVP....DS .M.......A ..R.Y..DEV ........N. .......... .R.......S .....

HY-20 .KS...IR.S .......... YSVP....DS .M.......A ..R.Y..DEV ........N. .......... .R.......S .....

Consensus .ks...irgs .......... %svp....ds .m.......a ..r.%..#ev .!......n. .......... .r.......s .....

**S5 Figure.** **Multiple alignment of the open reading frame 3 (ORF3) genomic region of the three rubus yellow net virus (RYNV) like isolates sequenced in this study relative to the previously identified sequences of RYNV-Ca (KF241951) and RYNV-BS (KM078034).** **A**, Multiple alignment of nucleotide sequences. Only nucleotides that differ from those of RYNV-Ca are shown. Identical nucleotides are indicated by dots. The aligned region corresponds to the nucleotides 6282-6840 of the complete nucleotides sequence of RYNV-Ca. **B**, Multiple alignment of amino acid sequences. Only amino acids that differ from those of RYNV-Ca are shown. Identical amino acids are indicated by dots.
