# Supplementary material for: Small-RNA analysis of pre-basic mother plants and conserved accessions of plant genetic resources for the presence of viruses
Source: PLoS One. 2019 Aug 7;14(8):e0220621. doi: 10.1371/journal.pone.0220621 (PMC6685626; doi:10.1371/journal.pone.0220621)
Supplement: S6 Fig — Only the nucleotides that differ from those of FN812699 are shown. Identical nucleotides are indicated by dots. The aligned region corresponds to nucleotides 265–1091 of FN812699. Wobble bases: K (G, T), R (A, G), Y (C, T), M (A, C), S (G, C), W (A, T), N (G, A, T, C). (DOCX) [file pone.0220621.s006.docx]

1 100

FN812699 CATCCCTTGG TCGAGCCGTT GAAAGGGATG CAACGAGTCA TTAATATAGG TCGAAAGAAT ATCATCAAGG ATTCATCATT AGCATCCGAT GCCGGTCGTA

RVCV-21 .......... .......... .......... .......... .......... .......... .......... .......... .......... ..........

RVCV-13 ......A... .......... A.....A... ..GA....T. .A........ .......... .....A.... .......... .......... ..........

RVCV-14 ......A... .......... A.....A... ..GA....T. .A........ .......... .....A.... .......... .......... ..........

RVCV-9-4 ......A... .......... A.....A... ..GA....T. .A........ .......... .....A.... .......... .......... ..........

RVCV-10 .....T..A. .......A.. A..G...... ........T. ....C..... ......AG.C .....A..A. ....C..C.. G...G.T... ........C.

RVCV-8 .....T..A. .......A.. A..G...... ........T. ....C..... ......AG.C .....A..A. ....C..C.. G...G.T... ........C.

RVCV-9-3 .....T..A. .......A.. A..G...... ........T. ....C..... ......AG.C .....A..A. ....C..C.. G...G.T... ........C.

RVCV-7_dps .....T..A. .......... A..G...... ........T. ....C..... ......RR.Y .....A..A. ....C..S.. G...K.T... ........C.

RVCV-8_dps .....TW.R. .......R.. A..R...... ........T. .W..Y..... ......RR.Y .....A..R. ....M..S.. R...K.T... ........Y.

RVCV-9_dps .....YA.R. .......R.. A..R..R... ..RAM...T. .W..Y..... .......... .....A..R. ....M..M.. R...K.Y... ........Y.

RVCV-10_dp ......A.R. .......R.. A..R..R... ..RM....T. .W..Y..... ......R..Y .....A..R. ....M..M.. R...K.Y... ........Y.

Consensus .......... .......... a.....g... ..acg...t. .......... .......a.. .....a.... .......... ....k..... ..........

101 200

FN812699 TGTTTAAGTT GATATTTTGC AGAGAGTATA AAGCAAAGAA CGGACATTAT CCTCCAGTGC TATCAGGACC AGAGTTGATT ATTACAGAAT TGTCGGAAGG

RVCV-21 .......... .......... .......... .......... .......... .......... .......... .......... .......... ..........

RVCV-13 .......... A......... .A......C. ....G..... ...G...... .........T .......... G......... .......... ....A.....

RVCV-14 .......... A......... .A......C. ....G..... ...G...... .........T .......... G......... .......... ....A.....

RVCV-9-4 .......... A......... .A......C. ....G..... ...G...... .........T .......... G......... .......... ....A.....

RVCV-10 .......... .C........ .A...A..C. .......... T......... .....G..A. .......... ....C....A .....T...C .A..A..G..

RVCV-8 .......... .C........ .A...A..C. .......... T......... .....G..A. .......... ....C....A .....T...C .A..A..G..

RVCV-9-3 .......... .C........ .A...A..C. .......... T......... .....G..A. .......... ....C....A .....T...C .A..A..G..

RVCV-7_dps .......... .C........ .A...R..Y. ....R..... Y......... .....R..R. .......... R...Y....W .....W...Y .R..A..R..

RVCV-8_dps .......... RM.......Y .A...R..Y. ....R..... Y..R...... .....R..RY .......... R...Y....W .....W...Y .R..A..R..

RVCV-9_dps .......... RM........ .A...R..C. ....R..... Y..R...... .....R..RY .......... R...Y..... .....W...Y .R..A..R..

RVCV-10_dp .......... RM........ .A...R..C. ....R..... ...R...... .....R..RY .......... R...Y....W .....W...Y .R..A..R..

Consensus .......... .........c .a...r..c. ....r..... .......... .....r..r. .......... r...y..... .....w...y .r..a..r..

201 300

FN812699 GGACCACAGA GCAACAAGTA AGAACGGACA CCCGTTGAGT GATTGGGATC GTGTGAAGTT CAAACAGCTG TTCCAACTAC CGGAAACTTT CAACTTATCC

RVCV-21 .......... .......... .......... .......... .......... .......... .......... .......... .......... ..........

RVCV-13 A.....T... .......... .A........ ...CC..... .......... .......... .......... ..T....... .......... ......G...

RVCV-14 A.....T... .......... .A........ ...CC..... .......... .......... .......... ..T....... .......... ......G...

RVCV-9-4 A.....T... .......... .A........ ...CC..... .......... .......... .......... ..T....... .......... ......G...

RVCV-10 A..T...... .......... .A..T..... T..T.....C ..C.....C. ....T..A.. T.....A..C ......T... .A........ ...T..G..G

RVCV-8 A..T...... .......... .A..T..... T..T.....C ..C.....C. ....T..A.. T.....A..C ......T... .A........ ...T..G..G

RVCV-9-3 A..T...... .......... .A..T..... T..T.....C ..C.....C. ....T..A.. T.....A..C ......T... .A........ ...T..G..G

RVCV-7_dps A..Y...... .......... .R..Y..... Y..KY....Y ..Y.....Y. ....K..R.. Y.....R..S ..Y...Y... .R........ ...Y..R..S

RVCV-8_dps A..Y...... .......... .R..Y..... Y..K.....Y ..Y.....Y. ....K..R.. Y.....R..S ..Y...Y... .R........ ...Y..R..S

RVCV-9_dps A..Y..Y... .......... .A..Y..... Y..YY....Y ..Y......Y ....K..R.. Y.....R..S ..Y...Y... .R........ ...Y..G..S

RVCV-10_dp A..Y..Y... .......... .A..Y..... Y..YY....Y ..Y.....Y. ....K..R.. Y.....R..S ..Y...Y... .R........ ...Y..G..S

Consensus a..y..c... .......... .a..y..... y........y ..y......c ....k..r.. y.....r..s ..y...y... .r........ ...y..g..s

301 400

FN812699 ATGATTGTTG CAGATAAGTC TATCTCACCC ACTAGATCAG AGCTTAGAAC GCTCATCAGG ACAAAGAGAA CAGTCATGAG TCCAGACAAG AGAAGAGGTG

RVCV-21 .......... .......... .......... .......... .......... .......... .......... .......... .......... ..........

RVCV-13 .......... .......A.. .......... .......... .A.....G.. A......C.. ..G....... .......... ...T...... ..........

RVCV-14 .......... .......A.. .......... .......... .A.....G.. A......C.. ..G....... .......... ...T...... ..........

RVCV-9-4 .......... .......A.. .......... .......... .A.....G.. A......C.. ..G....... .......... ...T...... ..........

RVCV-10 .......... .T........ ...A.....T ..C....... .......... A..T...... .......... .......... C..T...... ..G.....A.

RVCV-8 .......... .T........ ...A.....T ..C....... .......... A..T...... .......... .......... C..T...... ..G.....A.

RVCV-9-3 .......... .T........ ...A.....T ..C....... .......... A..T...... .......... .......... C..T...... ..G.....A.

RVCV-7_dps .......... .W........ ...M.....Y ..Y....... .R........ A..Y...M.. ..R....... .......... C..T...... ..R.....W.

RVCV-8_dps .......... .W........ ...M.....Y ..Y....... .R........ A..Y...M.. ..R....... .......... C..T...... ..R.....W.

RVCV-9_dps .......... .W.....R.. ...S.....Y ..Y....... .R.....R.. A..Y...M.. ..R....... .......... Y..T...... ..R.....W.

RVCV-10_dp .......... .W.....R.. ...M.....Y ..Y....... .R.....R.. A..Y...M.. ..R....... .......... Y..T...... ..R.....W.

Consensus .......... .w.....g.. .........y ..y....... .r.....a.. a..y...m.. ..r....... .......... ...t...... ..r.....w.

401 500

FN812699 TCAAGAGATG GTTGGAAGAT ACCACTCTAA ATCCTCGAGA ATTTTTGAAT TCGGTTAACG AAGGATTGTT CCCTGATGAT CATAAGGTGA TAGGGCTCAC

RVCV-21 .......... .........C .......... .....A.... .......... .......... .......... .......... .......... ..........

RVCV-13 .......... .........C .....C.... .C........ ...CC..... ..A..C.... ....G..A.. .......... .......... ..........

RVCV-14 .......... .........C .....C.... .C........ ...CC..... ..A..C.... ....G..A.. .......... .......... ..........

RVCV-9-4 .......... .........C .....C.... .C........ ...CC..... ..A..C.... ....G..A.. .......... .......... ..........

RVCV-10 .T........ .C.A.....C ..T....... .C..C..... ...C...G.. ..A....... .G........ .......... ........A. .......G..

RVCV-8 .T........ .C.A.....C ..T....... .C..C..... ...C...G.. ..A....... .G........ .......... ........A. .......G..

RVCV-9-3 .T........ .C.A.....C ..T....... .C..C..... ...C...G.. ..A....... .G........ .......... ........A. .......G..

RVCV-7_dps .Y........ .Y.R..R..C ..Y....... .C..Y..... ...CY..R.. ..A..Y..Y. .R..R..... .......... ........R. .......S..

RVCV-8_dps .Y........ .Y.R..R..C ..Y....... .C..Y..... ...CY..R.. ..A..Y..Y. .R..R..... .......... ........R. .......S..

RVCV-9_dps .Y........ .Y.R.....C ..Y..C.... .C..Y..... ...CY..R.. ..A..Y.... .R..R..R.. .......... .......... .......S..

RVCV-10_dp .Y........ ...R.....C .....Y.... .C..Y..... ...CY..R.. ..A..Y.... ....R..R.. .......... .......... .......S..

Consensus .y........ ...r..a..C .....t.... .c..y..... ...cy..r.. ..a..y..c. ....r..g.. .......... ........g. .......s..

501 600

FN812699 ACCAAAAGAG AGGGAACTAA ACCCCACACC TCGGATGTTT GCGCTAATGT CACATCTCTT GAGAGTGTAC GTTGTATTAA CGGAGCAGTT GATATCAGAC

RVCV-21 .......... .......... .......... .......... .....G.... .......... ......A... .......... .T........ ..........

RVCV-13 .......... ..A....... .......... .A........ .......... .......... .......... ..C....... .T........ ..........

RVCV-14 .......... ..A....... .......... .A........ .......... .......... .......... ..C....... .T........ ..........

RVCV-9-4 .......... ..A....... .......... .A........ .......... .......... .......... ..C....... .T........ ..........

RVCV-10 .........A .....GT.G. .T..T..... ...C...... ..T....... .......... AC.G..A... ..C...C.G. .C..A..... ..........

RVCV-8 .........A .....GT.G. .T..T..... ...C...... ..T....... .......... AC.G..A... ..C...C.G. .C..A..... ..........

RVCV-9-3 .........A .....GT.G. .T..T..... ...C...... ..T....... .......... AC.G..A... ..C...C.G. .C..A..... ..........

RVCV-7_dps .........R ..R..RT.G. .Y..Y..R.. .M.S...... ..K....... ....Y..... RM.R..R..Y ..C...Y.R. .Y..A..... ..........

RVCV-8_dps .........R ..R..RT.G. .Y..Y..R.. .M.S...... ..K....... ....Y..... RM.R..R..Y ..C...Y.R. .Y..A..... ..........

RVCV-9_dps .......... ..A...S..R .Y..YM.M.. .AS.M..... ...Y...... .......... .......W.. ..C...WY.R MYSRRMM... .........M

RVCV-10_dp .......... ..A..STWGR CY..T..... YAS....... ...YY..... .......... ......R... ..C...Y..R MTS.R..... K.....MR.M

Consensus .........g ......ttga ay...a.a.. t.gga..... ..gct..... ....t..... ga.a...t.c ..c....taa c.ga.ca... g.....ag.c

601 700

FN812699 CACGTCTTAA AATATTTTCC CCAGATAACA ATGACAGATA CCTTGCTAGA TTTGACCAAG AAGATGTACT CAACCGTCAA ACATCAATCT ATTCAGAACC

RVCV-21 .......... .......... .......... .......... .......... .......... .......... .......... .......... ..........

RVCV-13 ..T..T.... .G........ .......... ........C. .T........ .......... .......... .G........ .......... ..........

RVCV-14 ..T..T.... .G........ .......... ........C. .T........ .......... .......... .G........ .......... ..........

RVCV-9-4 ..T..T.... .G........ .......... ........C. .T........ .......... .......... .G........ .......... ..........

RVCV-10 ..TA....G. .......C.. ...A...... .....G.... .TC.AT.G.. ......A... ..A....... ....A..T.. .........C ........TA

RVCV-8 ..TA....G. .......C.. ...A...... .....G.... .TC.AT.G.. ......A... ..A....... ....A..T.. .........C ........TA

RVCV-9-3 ..TA....G. .......C.. ...A...... .....G.... .TC.AT.G.. ......A... ..A....... ....A..T.. .........C ........TA

RVCV-7_dps ..TR....R. .......Y.. ...A...... .....R..Y. .TC.RY.R.. ......A... ..R....... .R..M..Y.. .........Y ........YM

RVCV-8_dps ..TR.Y..R. .......C.. ...R...... .....R..Y. .TY.RY.R.. ......A... ..R....... .G..M..Y.. .........Y ........YM

RVCV-9_dps ..T.WTY..R .RWWW..... ..M...T..M ..KRMM.RMW MY.Y.M.W.. W..K.M...R ......K... YS......W. .......... M........Y

RVCV-10_dp .MT.WTY..G .RWWW..... ......T..M .....M.RMW MY.Y.M.... W..K.M...A .......... YS......T. .......... .....C...Y

Consensus .atgt.t.aa .atat..t.. ..ag..a..a ..gac..a.a ctttg..... t..g.cc..g ..g...t... c...c..ca. .........t a....g..c.

701 800

FN812699 GACAGAGAGG AAAGGATAAC CTCTGGGCTT CCAGGGTTAT TTGCATGTCA TTAGATTTCG AGAAATGGAA TGGGCATATG AGAAAAGAGA TGACATCAGG

RVCV-21 .......... .........T ..A....... .......... .......... .......... .......... .......... .......... ..........

RVCV-13 .G........ .........T .......... .......... .......... ........T. .......... ...T...... ..G....... ..........

RVCV-14 .G........ .........T .......... .......... .......... ........T. .......... ...T...... ..G....... ..........

RVCV-9-4 .G........ .........T .......... .......... .......... ........T. .......... ...T...... ..G....... ..........

RVCV-10 ....A..G.. ...A.....T ........A. .T..A..... A........C C.......T. ....G..... C.....C... ..G....... ..........

RVCV-8 ....A..G.. ...A.....T ........A. .T..A..... A........C C.......T. ....G..... C.....C... ..G....... ..........

RVCV-9-3 ....A..G.. ...A.....T ........A. .T..A..... A........C C.......T. ....G..... C.....C... ..G....... ..........

RVCV-7_dps ....R..R.. ...R....MT ..S.....W. .T..R..... A........M Y.......T. ....G..... Y..K..Y... ..R....... ..........

RVCV-8_dps .......R.. ...R....MT ........A. .Y..R..... A........M Y.......T. ....G..... Y..K..Y... ..R....... ..........

RVCV-9_dps RGM....... ...R.....T .......... ....R..... W......... Y.......T. .......... Y..K..Y... ..G....... ..........

RVCV-10_dp RGM....... .........T .......... .T..R..... .........M Y.......T. ....G..... ...T...... ..G....... ..........

Consensus gac.g..a.. ........aT ..c.....t. ....r..... .......... y.......t. ....g..... .......... ..g....... ..........

801 827

FN812699 GGTTTTCACA GCGTTAGGGG ACTTGTT

RVCV-21 .......... .......... .......

RVCV-13 A......... ..T....... ....A..

RVCV-14 A......... ..T....... ....A..

RVCV-9-4 A......... ..T....... ....A..

RVCV-10 A......... ........A. ....A..

RVCV-8 A......... ........A. ....A..

RVCV-9-3 A......... ........A. ....A..

RVCV-7_dps A......... ........A. ....A..

RVCV-8_dps A......... ........A. ....A..

RVCV-9_dps A......... ..K.....R. ....A..

RVCV-10_dp A......... ..T.....A. ....A..

Consensus a......... ..g.....a. ....a..

**S6 Fig. Nucleotide sequence alignment of the previously published L polymerase gene fragment of RVCV (database number FN812699) and seven RVCV isolates sequenced in this study**. Only the nucleotides that differ from those of FN812699 are shown. Identical nucleotides are indicated by dots. The aligned region corresponds to nucleotides 265−1091 of FN812699. Wobble bases: K (G, T), R (A, G), Y (C, T), M (A, C), S (G, C), W (A, T), N (G, A, T, C).
