# Supplementary material for: Small-RNA analysis of pre-basic mother plants and conserved accessions of plant genetic resources for the presence of viruses
Source: PLoS One. 2019 Aug 7;14(8):e0220621. doi: 10.1371/journal.pone.0220621 (PMC6685626; doi:10.1371/journal.pone.0220621)
Supplement: S1 Table — (DOCX) [file pone.0220621.s007.docx]

**S1 Table. Plant samples from pre-basic mother plants, breeding lines, and indicator plants from Luke-Laukaa (GEN17 to GEN20), samples from field-grown *Rubus* plants of the genetic resource collection at Luke-Piikkiö and two samples of field grown *Ribes* plants (HXR1 and HXR2).**

| **Sample pool** | **Sample**  **number** | **Sample ID**  **(Luke Laukaa)** | **Plant species** | **Cultivar/line** |
| --- | --- | --- | --- | --- |
| GEN17 | 14555 | 14555 | *Fragaria x ananassa* | Bounty^1^ |
|  | 14556 | 14556 | *Fragaria x ananassa* | Bounty^1^ |
|  | 14557 | 14557 | *Fragaria x ananassa* | Bounty^1^ |
|  | 14558 | 14558 | *Fragaria x ananassa* | Bounty^1^ |
|  | 14559 | 14559 | *Fragaria x ananassa* | Bounty^1^ |
|  | 14560 | 14560 | *Fragaria x ananassa* | Bounty^1^ |
|  | 14561 | 14561 | *Fragaria x ananassa* | Honeoye^1^ |
|  | 14562 | 14562 | *Fragaria x ananassa* | Honeoye^1^ |
|  | 14563 | 14563 | *Fragaria x ananassa* | Honeoye^1^ |
|  | 14564 | 14564 | *Fragaria x ananassa* | Honeoye^1^ |
|  | 14565 | 14565 | *Fragaria x ananassa* | Honeoye^1^ |
|  | 14566 | 14566 | *Fragaria x ananassa* | Jonsok^1^ |
|  | 14567 | 14567 | *Fragaria x ananassa* | Jonsok^1^ |
|  | 14568 | 14568 | *Fragaria x ananassa* | Jonsok^1^ |
|  | 14569 | 14569 | *Fragaria x ananassa* | Jonsok^1^ |
|  | 14570 | 14570 | *Fragaria x ananassa* | Kaunotar^1^ |
|  | 14571 | 14571 | *Fragaria x ananassa* | Kaunotar^1^ |
|  | 14572 | 14572 | *Fragaria x ananassa* | Kaunotar^1^ |
|  | 14573 | 14573 | *Fragaria x ananassa* | Korona^1^ |
|  | 14574 | 14574 | *Fragaria x ananassa* | Korona^1^ |
|  | 14575 | 14575 | *Fragaria x ananassa* | Kulkuri^1^ |
|  | 14576 | 14576 | *Fragaria x ananassa* | Kulkuri^1^ |
|  | 14577 | 14577 | *Fragaria x ananassa* | Polka^1^ |
|  | 14578 | 14578 | *Fragaria x ananassa* | Polka^1^ |
|  | 14579 | 14579 | *Fragaria x ananassa* | Polka^1^ |
|  | 14580 | 14580 | *Fragaria x ananassa* | Polka^1^ |
|  | 14581 | 14581 | *Fragaria x ananassa* | Polka^1^ |
|  | 14582 | 14582 | *Fragaria x ananassa* | Polka^1^ |
|  | 14583 | 14583 | *Fragaria x ananassa* | Polka^1^ |
|  | 14584 | 14584 | *Fragaria x ananassa* | Ria^1^ |
|  |  |  |  |  |
| GEN18 | RIA | 14585 | *Fragaria x ananassa* | Ria^1^ |
|  | RIA | 14586 | *Fragaria x ananassa* | Ria^1^ |
|  | SS | 14587 | *Fragaria x ananassa* | Senga Sengana^1^ |
|  | SS | 14588 | *Fragaria x ananassa* | Senga Sengana^1^ |
|  | SS | 14589 | *Fragaria x ananassa* | Senga Sengana^1^ |
|  | SUVE | 14590 | *Fragaria x ananassa* | Suvetar^1^ |
|  | SUVE | 14591 | *Fragaria x ananassa* | Suvetar^1^ |
|  | SUVE | 14592 | *Fragaria x ananassa* | Suvetar^1^ |
|  | TTA-786 | 14593 | *Fragaria x ananassa* | Lumotar^1^ |
|  | TTA-786 | 14594 | *Fragaria x ananassa* | Lumotar^1^ |
|  | TTA-786 | 14595 | *Fragaria x ananassa* | Lumotar^1^ |
|  | VALO | 14596 | *Fragaria x ananassa* | Valotar^1^ |
|  | VALO | 14597 | *Fragaria x ananassa* | Valotar^1^ |
|  | VALO | 14598 | *Fragaria x ananassa* | Valotar^1^ |
|  | X-4 | 14599 | *Fragaria vesca* | U.C-5^2^ |
|  | X-4 | 14600 | *Fragaria vesca* | U.C-5^2^ |
|  | X-5 | 14601 | *Fragaria vesca* | U.C-6^2^ |
|  | X-5 | 14602 | *Fragaria vesca* | U.C-6^2^ |
|  | X-7 | 14603 | *Fragaria virginiana* | U.C-11^2^ |
|  | X-7 | 14604 | *Fragaria virginiana* | U.C-11^2^ |
|  | X-15 | 14605 | *Rubus occidentalis* | Cumberland^2^ |
|  | X-16 | 14607 | *Rubus idaeus* | Malling Delight^2^ |
|  | X-16 | 14608 | *Rubus idaeus* | Malling Delight^2^ |
|  | X-17 | 14609 | *Rubus idaeus* | Malling Landmark^2^ |
|  | X-17 | 14610 | *Rubus idaeus* | Malling Landmark^2^ |
|  | Z-13 | 14611 | *Rubus idaeus* | Z-13^3^ |
|  | Z-22 | 14612 | *Rubus idaeus* | Z-22^4,5^ |
|  | Z-17 | 14613 | *Rubus idaeus* | Z-17^6,5^ |
|  | 14743 | 14743 | *Fragaria x ananassa* | 146^7^ |
|  | 14744 | 14744 | *Fragaria x ananassa* | 227^7^ |
|  | 14573 | 14573 | *Fragaria x ananassa* | Korona^1^ |
|  |  |  |  |  |
| GEN19 | 51 | 14689 | *Rubus idaeus* | Jatsi^1^ |
|  | 53 | 14693 | *Rubus idaeus* | Maurin Makea^1^ |
|  | 54 | 14694 | *Rubus idaeus* | Maurin Makea^1^ |
|  | 55 | 14695 | *Rubus idaeus* | Muskoka^1^ |
|  | 56 | 14696 | *Rubus idaeus* | Muskoka^1^ |
|  | 57 | 14697 | *Rubus idaeus* | Muskoka^1^ |
|  | 58 | 14698 | *Rubus idaeus* | Ottawa^1^ |
|  | 61 | 14701 | *Rubus idaeus* | Takalan Herkku^1^ |
|  | 62 | 14702 | *Rubus idaeus* | Takalan Herkku^1^ |
|  | 63 | 14703 | *Rubus idaeus* | Takalan Herkku^1^ |
|  | 60b | 14700 | *Rubus idaeus* | Ottawa^1^ |
|  | 64 | 14704 | *Rubus idaeus* | Z-13^8^ |
|  | 65 | 14705 | *Rubus idaeus* | Z-21^5,6^ |
|  | 66 | 14706 | *Rubus idaeus* | Z-21^5,6^ |
|  | 67 | 14707 | *Rubus idaeus* | Z-21^5,6^ |
|  | 68 | 14708 | *Rubus idaeus* | Z-23^5,6^ |
|  | 69 | 14709 | *Rubus idaeus* | Z-23^5,6^ |
|  | 70 | 14710 | *Rubus idaeus* | Z-23^5,6^ |
|  | 71 | 14711 | *Rubus idaeus* | Z-23^5,6^ |
|  | 74 | 14607 | *Rubus idaeus* | Malling Delight^2^ |
|  | 75 | 14608 | *Rubus idaeus* | Malling Delight^2^ |
|  | 76 | 14609 | *Rubus idaeus* | Malling Landmark^2^ |
|  | 77 | 14610 | *Rubus idaeus* | Malling Landmark^2^ |
|  | 52 | 14692 | *Rubus idaeus* | Maurin Makea^1^ |
|  | 59 | 14699 | *Rubus idaeus* | Ottawa^1^ |
|  | 49 | 14687 | *Rubus x binatus* | Heisa^1^ |
|  | 50 | 14688 | *Rubus x binatus* | Heisa^1^ |
|  | 45 | 14683 | *Ribes uva-crispa* | Hinnonmäen Kelt.^1^ |
|  | 46 | 14684 | *Ribes uva-crispa* | Hinnonmäen Kelt.^1^ |
|  | 47 | 14685 | *Ribes uva-crispa* | Lepaan Punainen^1^ |
|  | 48 | 14686 | *Ribes uva-crispa* | Lepaan Punainen^1^ |
|  | 30 | 14668 | *Ribes nigrum* | Vilma^1^ |
|  | 31 | 14669 | *Ribes nigrum* | Vilma^1^ |
|  | 33 | 14671 | *Ribes nigrum* | Venny^1^ |
|  | 34 | 14672 | *Ribes nigrum* | Venny^1^ |
|  | 35 | 14673 | *Ribes nigrum* | Venny^1^ |
|  | 32 | 14670 | *Ribes nigrum* | Vilma^1^ |
|  | 72b | 14623 | *Ribes rubrum* | Piikkiön Helmi^1^ |
|  | 73b | 14624 | *Ribes rubrum* | Lepaan Valkea^1^ |
|  | 11 | 14649 | *Ribes nigrum* | Mikael^1^ |
|  | 4 | 14642 | *Ribes nigrum* | Mortti^1^ |
|  | 6 | 14644 | *Ribes nigrum* | Ola^1^ |
|  | 16 | 14654 | *Ribes nigrum* | Öjebyn^1^ |
|  |  |  |  |  |
| GEN20 | 2 | 14640 | *Ribes nigrum* | Hedda^1^ |
|  | 3 | 14641 | *Ribes nigrum* | Mortti^1^ |
|  | 7 | 14645 | *Ribes nigrum* | Marski^1^ |
|  | 8 | 14646 | *Ribes nigrum* | Marski^1^ |
|  | 9 | 14647 | *Ribes nigrum* | Marski^1^ |
|  | 10 | 14648 | *Ribes nigrum* | Marski^1^ |
|  | 12 | 14650 | *Ribes nigrum* | Mikael^1^ |
|  | 14 | 14652 | *Ribes nigrum* | Mikael^1^ |
|  | 15 | 14653 | *Ribes nigrum* | Öjebyn^1^ |
|  | 17 | 14655 | *Ribes nigrum* | Piikkiö 15^7^ |
|  | 18 | 14656 | *Ribes nigrum* | Piikkiö 15^7^ |
|  | 19 | 14657 | *Ribes nigrum* | Piikkiö 15^7^ |
|  | 20 | 14658 | *Ribes nigrum* | Piikkiö 15^7^ |
|  | 21b | 14659 | *Ribes rubrum* | Punainen Holl.^1^ |
|  | 23b | 14661 | *Ribes rubrum* | Katri^1^ |
|  | 24b | 14662 | *Ribes rubrum* | Katri^1^ |
|  | 25 | 14663 | *Ribes rubrum* | Punahilkka^1^ |
|  | 26 | 14664 | *Ribes rubrum* | Punahilkka^1^ |
|  | 27b | 14665 | *Ribes rubrum* | 90026058^7^ |
|  | 28b | 14666 | *Ribes rubrum* | 90026058^7^ |
|  | 29b | 14667 | *Ribes rubrum* | 90026058^7^ |
|  | 22b | 14660 | *Ribes rubrum* | Punainen Holl.^1^ |
|  | 36 | 14674 | *Ribes rubrum* | Piikkiön Helmi^1^ |
|  | 37 | 14675 | *Ribes rubrum* | Piikkiön Helmi^1^ |
|  | 38 | 14676 | *Ribes rubrum* | Piikkiön Helmi^1^ |
|  | 39b | 14677 | *Ribes rubrum* | Lepaan Valkea^1^ |
|  | 40b | 14678 | *Ribes rubrum* | Lepaan Valkea^1^ |
|  | 41b | 14679 | *Ribes rubrum* | Lepaan Valkea^1^ |
|  | 42b | 14680 | *Ribes rubrum* | Lepaan Valkea^1^ |
|  | 43b | 14681 | *Ribes rubrum* | Valkoinen Suomal.^1^ |
|  | 44 | 14682 | *Ribes rubrum* | Valkoinen Suomal.^1^ |
|  | 5 | 14643 | *Ribes nigrum* | Ola^1^ |

| **Sample**  **pool** | **Sample number** | **Sample ID**  **(Luke Laukaa)** | **Plant species** | **Cultivar/variety** |
| --- | --- | --- | --- | --- |
| HXR1 | 1 | 16004 | *Rubus idaeus* | Jenkka^9^ |
|  | 2 | 16005 | *Rubus idaeus* | Jenkka^9^ |
|  | 3 | 16006 | *Rubus idaeus* | Maurin Makea^9^ |
|  | 4 | 16007 | *Rubus idaeus* | Maurin Makea^9^ |
|  | 5 | 16008 | *Rubus idaeus* | RU20 Preussen^10^ |
|  | 6 | 16009 | *Rubus idaeus* | RU20 Preussen^10^ |
|  | 7 | 16010 | *Rubus x binatus* | RU53^9^ |
|  | 8 | 16011 | *Rubus x binatus* | RU53^9^ |
|  | 9 | 16012 | *Rubus x binatus* | RU54^9^ |
|  | 10 | 16013 | *Rubus x binatus* | RU54^9^ |
|  | 11 | 16014 | *Rubus idaeus* | RU168 Krusenbergs^10^ |
|  | 12 | 16015 | *Rubus idaeus* | RU168 Krusenbergs^10^ |
|  | 13 | 16016 | *Rubus x binatus* | RU55^9^ |
|  | 14 | 16017 | *Rubus x binatus* | RU55^9^ |
|  | 15 | 16018 | *Rubus idaeus f. chlorocarpus* | Keltainen Pisa^9^ |
|  | 16 | 16019 | *Rubus idaeus f. chlorocarpus* | Keltainen Pisa^9^ |
|  | 17 | 16020 | *Rubus nessensis* | Uusikirkko^9^ |
|  | 18 | 16021 | *Rubus nessensis* | Uusikirkko^9^ |
|  | 19 | 16022 | *Rubus idaeus x R. allegheniensis* | HY 6230^9^ |
|  | 20 | 16023 | *Rubus idaeus x R. allegheniensis* | HY 6230^9^ |
|  | 21 | 16024 | *Rubus idaeus* | RU25 Norna^10^ |
|  |  | |  |  |
| HXR2 | 22 | 16025 | *Rubus idaeus* | RU25 Norna^10^ |
|  | 23 | 16026 | *Rubus idaeus* | RU158 Hoolin kanta^10^ |
|  | 24 | 16027 | *Rubus idaeus* | RU158 Hoolin kanta^10^ |
|  | 25 | 16028 | *Rubus idaeus* | RU159 Ranta, Kaukonen^9^ |
|  | 26 | 16029 | *Rubus idaeus* | RU159 Ranta, Kaukonen^9^ |
|  | 27 | 16030 | *Rubus idaeus* | RU24, Heija^10^ |
|  | 28 | 16031 | *Rubus idaeus* | RU24, Heija^10^ |
|  | 29 | 16032 | *Rubus idaeus* | RU156 Ojanperä, Kaukonen^9^ |
|  | 30 | 16033 | *Rubus idaeus* | RU156 Ojanperä, Kaukonen^9^ |
|  | 31 | 16034 | *Rubus allegheniensis* | Majestät^10^ |
|  | 32 | 16035 | *Rubus allegheniensis* | Majestät^10^ |
|  | 33 | 16036 | *Rubus idaeus* | RU18 Heisa^9^ |
|  | 34 | 16037 | *Rubus idaeus* | RU18 Heisa^9^ |
|  | 35 | 16038 | *Rubus idaeus x R. allegheniensis* | HY 71029^9^ |
|  | 36 | 16039 | *Rubus idaeus x R. allegheniensis* | HY 71029^9^ |
|  | 37 | 16040 | *Rubus idaeus* | Ville^9^ |
|  | 38 | 16041 | *Rubus idaeus* | Ville^9^ |
|  | 39 | 16002 | *Rubus idaeus* | Indian Summer^9^ |
|  | 40 | 16003 | *Rubus idaeus* | Indian Summer^9^ |
|  | 41 | 16042 | *Ribes nigrum* | Mara |
|  | 42 | 16043 | *Ribes rubrum* | Valkoinen Suomalainen |

^1^ A pre-basic mother plant

^2^ An indicator plant of *Fragaria* or *Rubus*

^3^ A breeding line of *Rubus*, which is known to contain *Raspberry bushy dwarf virus*

^4^ An unnamed propagation line of yellow *Rubus*

^5^ Suspected virus infection

^6^ A breeding line of *Rubus*

^7 A^ breeding line of *Fragaria* or *Ribes*, not named cultivar

^8^ A virus free breeding line of *Rubus* cultivar Z-13 (the same breeding line as in index 3 but virus free).

^9^ A clone selected to the long-term preservation

^10^ An old Finnish *Rubus* clone, not selected to the long term preservation
