# Supplementary material for: Small-RNA analysis of pre-basic mother plants and conserved accessions of plant genetic resources for the presence of viruses
Source: PLoS One. 2019 Aug 7;14(8):e0220621. doi: 10.1371/journal.pone.0220621 (PMC6685626; doi:10.1371/journal.pone.0220621)
Supplement: S2 Table — (DOCX) [file pone.0220621.s008.docx]

**S2 Table. Primers used in the study.**

| **Primer** | **Sequence (5’−3’)** |  | **Nucleotide position^1^** | **Target** | **Origin** |
| --- | --- | --- | --- | --- | --- |
| **RBDV L3** | CGTCGACGGCSCCGCCCACCACA | | 1835−1857 | RBDV | Kokko et al. 1996a |
| **RBDV U2** | TTCATCCTCCAAATCTCAGCAAC | | 1613−1635 | RBDV | Kokko et al. 1996a |
| **BRNV F1** | TATCTCGTACTCCACCAAC | | 3967−3985 | BRNV | Susi et al. 2018 |
| **BRNV R1** | TGGTTCAATGCCTTTACCC | | 4606−4624 | BRNV | this work |
| **BRNV R2** | CTATAGACCTAGGGCACC | | 5408−5426 | BRNV | Susi et al. 2018 |
| **RVCV F1** | ACCAATATCCATCATCTGAC | | 214−233 | RVCV | this work |
| **RVCV R1** | GTAACACTCCTTGAATATATC | | 1123−1143 | RVCV | this work |
| **RVCV F2** | AGAGAGGAAAGGATAATCTCTG | | 968−989 | RVCV | this work |
| **RVCV R2** | GTCTAACAGTTCCCTCAACC | | 2036−2055 | RVCV | this work |
| **RVCV F3** | GCAGAAAGGCCTAACAACG | | 1860−1878 | RVCV | this work |
| **RVCV R3** | TTGATAACAAGTAACTCGAGC | | 2990−3010 | RVCV | this work |
| **RYNV F1** | TCTTCCARAGRAAGATGGACC | | 6036−6056 | RYNV | this work |
| **RYNV R1** | CCYTTKATRTGTTCGAACTTC | | 6859−6879 | RYNV | this work |
| **RYNV F2** | TMAAGAAGATAGCYGAGGTGG | | 6282−6302 | RYNV | this work |
| **RYNV F3?** | TAAGGTGGTTAGGTTTTTGTG | | 6810−6830 | RYNV | this work |
| **RYNV R2** | AAGGAAAGTCTAGAGAGGTTG | | 516−536 | RYNV | this work |
| **GVBaV F1** | GGATGGGGAGGAGTGTGC | | 6664−6681 | GVBaV | this work |
| **GVBaV R1** | ATAAGATTCCCTGACTCAGG | | 7311−7332 | GVBaV | this work |
| **BRV F1** | GTCTACCTGCTTCCGAGCCT | | 5724−5742 | BRV | this work |
| **BRV R1** | CTAGCGCTTGCAACGCTAGT | | 6215−6235 | BRV | this work |

^1^ BRNV, black raspberry necrosis virus; BRV, blackcurrant reversion virus; GVBaV, gooseberry vein banding associated virus; RBDV, raspberry bushy dwarf virus; RVCV, raspberry vein chlorosis virus; RYNV, *Rubus* yellow net virus.

^2^ The following reference sequences were used: RBDV (NC_003740.1), BRNV (NC_008183.1), RVCV (FN812699.2), RYNV (KF241951.1), GVBaV (NC_018105.1), and BRV (NC_003502.1)
